# Modeling the distribution of pine wilt disease in China using the ensemble models MaxEnt and CLIMEX

**DOI:** 10.1002/ece3.70277

**Published:** 2024-09-19

**Authors:** Lin Chen, Wenxiong Lu, Byron B. Lamont, Yu Liu, Pujie Wei, Weixing Xue, Zixuan Xiong, Li Tang, Yongjian Wang, Pengcheng Wang, Zhaogui Yan

**Affiliations:** ^1^ College of Horticulture and Forestry Sciences/Hubei Engineering Technology Research Center for Forestry Information Huazhong Agricultural University Wuhan China; ^2^ Ecology Section, School of Molecular and Life Sciences Curtin University Perth Western Australia Australia; ^3^ College of Agriculture and Animal Husbandry Qinghai University Xining China

**Keywords:** beetle vector, CLIMEX, MaxEnt, *Monochamus*, pine wilt disease, pinewood nematode

## Abstract

Pine wilt disease (PWD) is a devastating plant disease caused by the pinewood nematode (PWN, *Bursaphelenchus xylophilus*) that is transmitted by several beetle species in the genus, *Monochamus*. Once present, the disease is difficult to control. Prevention rather than control is regarded as an effective strategy for PWD management. Central to this prevention strategy is the ability to predict the potential distribution of the disease. Here, we employed an integrated MaxEnt and CLIMEX approach to model the potential distribution of PWD under various climate‐change scenarios. Our results indicate that rising temperatures and lower humidity under climate change will render some of the northern regions of China more suitable for the nematode and these beetles, causing the gradual northward movement of PWD. Furthermore, suitable habitats for three pine species, *Pinus massoniana*, *P. taiwanensis* and *P. shurbergia*, overlap with PWN and *Monochamus*, suggesting that these three species are potentially at high risk of PWD. Thus, PWD management should target the northern regions of China and the three pine species that are most susceptible to PWD.

## Graphical abstract

Rising temperatures and drought under climate change will render some areas of northern China suitable for pine wood nematode and its insect vector. Pine wilt disease (PWD) will gradually move northward. *Pinus massoniana*, *P. taiwanensis* and *P. shurbergia* are at high risk of PWD.

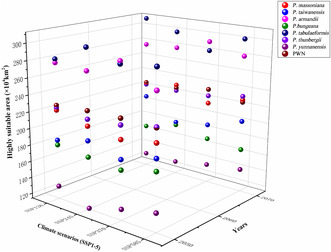

## 1 INTRODUCTION

Pine wilt disease (PWD) is a devastating plant disease caused by the pinewood nematode (PWN, *Bursaphelenchus xylophilus*, family Parasitaphelenchidae) that affects pine species worldwide (Cheng et al., 1983; Rutherford & Webster, 1987; Steiner & Buhrer, 1934). PWD results from tissue damage by the microscopic eelworm transmitted by the beetles, *Monochamus alternatus*, *M. saltuarius*, *M. galloprovincialis*, and *M. carolinensis*, order Coleoptera, family Cerambycidae (Akbulut & Stamps, 2012; Chen et al., 2020; Firmino et al., 2017; Nakayama & Togashi, 2023; Wang, Wang, et al., 2022). In Portugal, *M. galloprovincialis* is the only vector (Firmino et al., 2017). In China, *M. alternatus* was initially considered the only vector (Li et al., 2020). However, *M. saltuarius* was also found to be a vector of PWN in 2020 (Gao, Liu, Li, et al., 2023). Originating in North America (Steiner & Buhrer, 1934), PWN has spread rapidly across Asia, including Japan, Korea, and China, later extending to Europe, and precipitating a PWD pandemic (Cheng et al., 1983; Robinet et al., 2011; Rutherford & Webster, 1987; Yano, 1913; Yi et al., 1989).

Pine wilt disease has emerged as a major concern in Asia, causing extensive damage to pine forests in Japan, Korea, and China (Ryss et al., 2011). In Japan, Pine wilt disease has wreaked havoc since its discovery in 1905, affecting the entire country except the northernmost region (Kishi, 1995). In Korea, PWD was first reported in 1988, and the damaged area continues to escalate (Choi et al., 2019; Nguyen et al., 2017; Syifa et al., 2020). Similarly, in China, since its first identification in 1982 (Cheng et al., 1983), the disease has swiftly spread across 18 provinces, leading to widespread pine tree mortality by 2022 (Wang, Peng, et al., 2022; Wang, Wang, et al., 2022; Zhao, 2008). The lack of effective control measures has made the situation worse, as the disease spreads to new areas across China (Gao, Liu, Zhao, & Cui, 2023).

The symptoms of PWD include wilting, yellowing, and browning of pine needles, which lead to tree death (Mamiya, 1983). From first infection to tree mortality, the disease can develop within a few weeks or months, resulting in significant economic losses (Roy et al., 2014; Zhao et al., 2020). According to the Chinese National Forestry Bureau (https://www.forestry.gov.cn/), the disease has affected over 2.5 million hectares of forest in China, resulting in total financial losses of up to 20 billion Ren Min Bi (RMB).

Apart from causing economic losses, the disease can also have detrimental ecological consequences, including decreased carbon sequestration and soil erosion (Brockerhoff et al., 2017) and the loss of biodiversity, as pine forests are habitats for many other species (Zhao et al., 2020). Once present, PWD is difficult and expensive to control (Shin, 2008; Wu et al., 2020). Prevention rather than control has been called for as an effective strategy for PWD management. Central to this prevention strategy is the ability to predict the potential distribution of the disease.

Species distribution modeling, leveraging algorithms like MaxEnt, offers a powerful tool to estimate the relationship between species occurrence and environmental factors (Puchałka et al., 2023; Yan et al., 2018). Widely applied in predicting the potential distribution of invasive species or diseases; this method has been employed in PWD predictions (Ouyang et al., 2022; Tang et al., 2021; Wang, Peng, et al., 2022; Wang, Wang, et al., 2022; Yang et al., 2023; Yoon et al., 2023). Earlier studies have mainly focused on the biology and ecology of the PWN and *Monochamus* species (Akbulut & Stamps, 2012; Rutherford et al., 1990). However, more recent studies in Japan (Mukasyaf et al., 2021), Korea (Yoon et al., 2023), Europe (De La Fuente & Saura, 2021), and America (Atkins et al., 2021) have been conducted on the management of PWD and on techniques for its early detection and control. Techniques such as remote sensing and sanitary felling and traps with chemical lures have been used for early detection and for beetle control (Ikenaka et al., 2019; Long et al., 2023).

Climate factors are important to the distribution and spread of plant diseases (Rutherford et al., 1990; Rutherford & Webster, 1987). The CLIMEX is a modeling software developed for predicting the geographic distribution of plant and animal species based on the environmental requirements of the species (Byeon et al., 2018; Kriticos et al., 2015). For instance, the model has been used to predict the potential distribution of plant and animal species by using combinations of climatic factors such as temperature and altitude and biological factors such as competition and reproduction (Byeon et al., 2018; Kriticos et al., 2012; Taylor et al., 2012). The model has been used to determine the influence of various climate factors on the spread of PWD (Song & Xu, 2006; Yoon et al., 2023). Several studies have examined the impact of climate change on the spread of PWD in China (Ouyang et al., 2022; Wang, Peng, et al., 2022; Wang, Wang, et al., 2022) and in other countries (Aslam et al., 2021; Hirata et al., 2017; Ikegami & Jenkins, 2018). Results show that temperature and drought play key roles in the occurrence and development of PWD. Hot and arid conditions have been found to promote the spread of disease, raising concerns that climate change could lead to changes in the potential distribution area of PWD. As temperature and precipitation change under climate change, the areas suitable for PWD may change, and certain previously unsuitable areas for its distribution may become suitable (Hirata et al., 2017).

**FIGURE 1:**
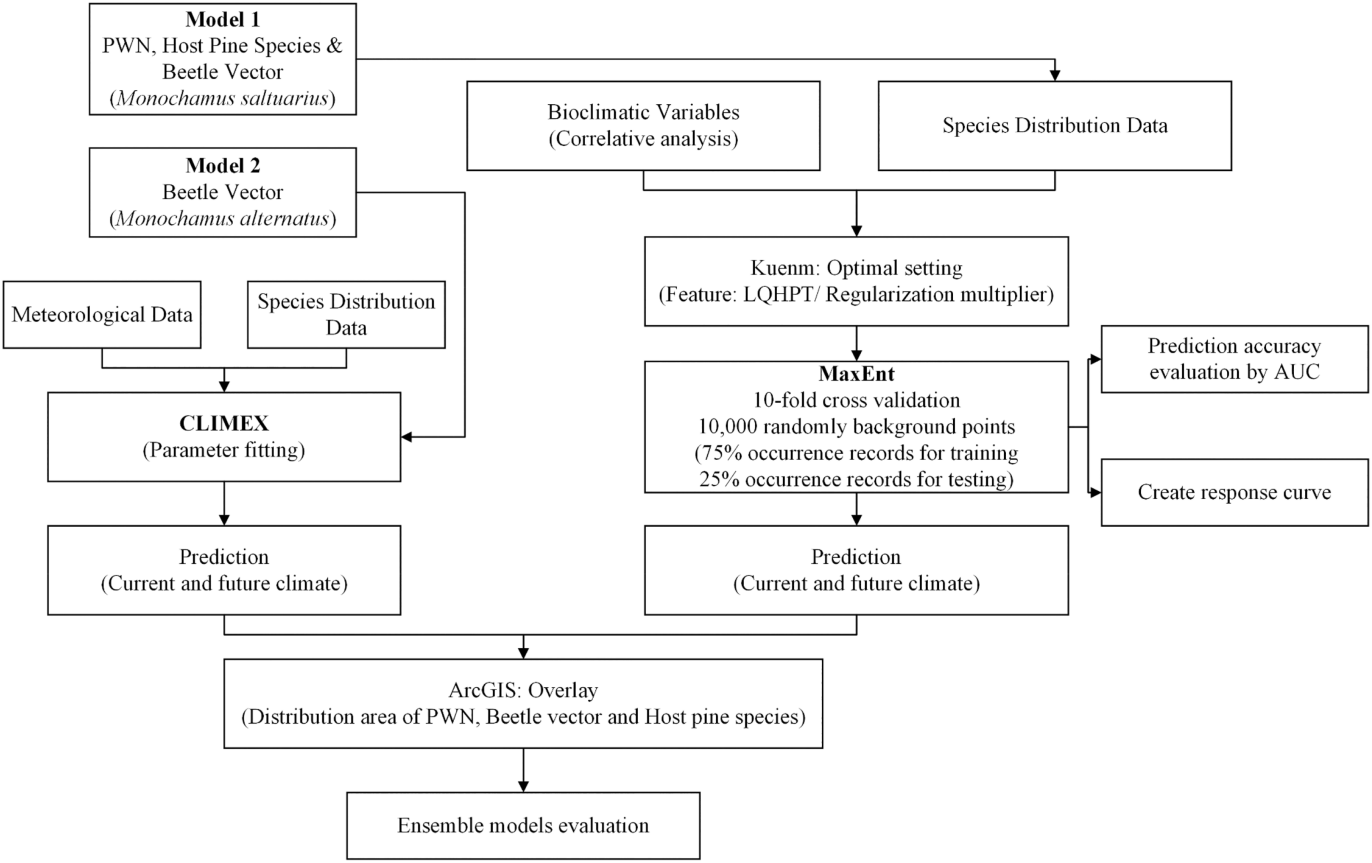
Flowchart to understand the overall analysis: Potential distribution of the disease through the overlap of the three participants: Pine species, nematode and beetle vector. AUC, area under the receiver operating characteristic curve; PWN, pinewood nematode.

Most existing studies on PWD have used single‐model approaches (Gao, Liu, Zhao, & Cui, 2023; Jeschke & Strayer, 2008; Tang et al., 2021; Wang, Peng, et al., 2022; Wang, Wang, et al., 2022) that may not capture the full complexity of the problem. Here, we aimed to determine the interrelationship between ecological and environmental factors and the spread of PWD under various climate change scenarios. We undertook the integrated modeling approach by using the MaxEnt model to predict the potentially suitable areas of pine species, PWN and *M. saltuarius* and the CLIMEX model to predict the potential distribution of *M. alternatus*. The overlapping of the three participants, pine species, nematode and its beetle vector, is used to determine the potential distribution of the disease (Figure 1). Our approach was not only able to use the occurrence data of pine species and the PWN but also data on climatic factors under climate change. The predicted distribution of the PWD can be used as a part of the disease prevention strategy under various climate change scenarios.

## 2 MATERIALS AND METHODS

### 2.1 Distribution data

Distribution data for PWD at the township level from 2015 to 2022 were downloaded from the State Forestry and Grassland Administration of China (https://www.forestry.gov.cn/) and from published data of the forestry departments of 19 provinces (municipalities) in China. The 1:4,000,000 map (http://www.cehui8.com/3S/GIS20130702/205.html) was used as the base map for all analyses in this study.

**FIGURE 2:**
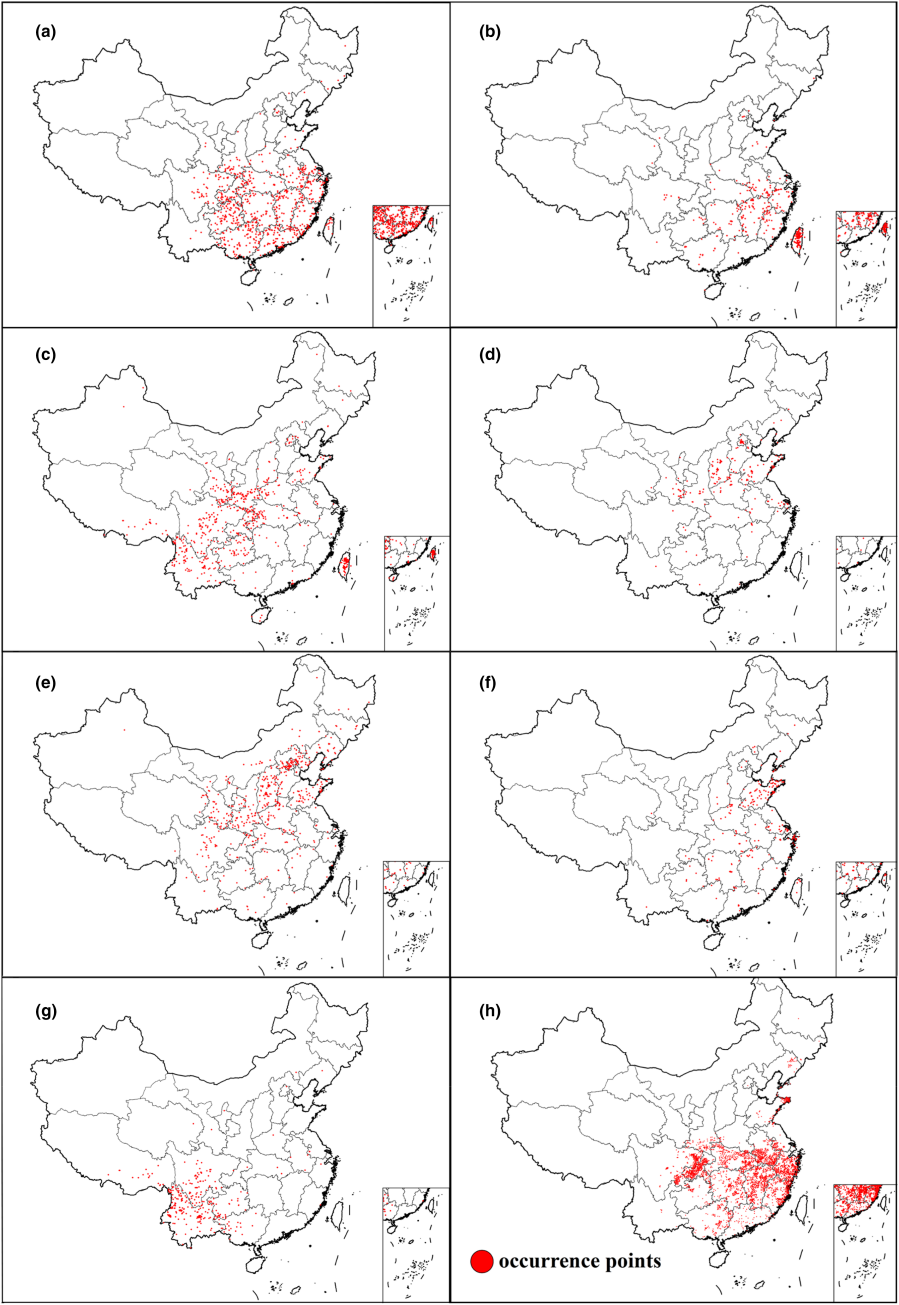
Current distribution points of seven pine species and pinewood nematode. (a) *Pinus massoniana*, (b) *P. taiwanensis*, (c) *P. armandii*, (d) *P. bungeana*, (e) *P. tabuliformis*, (f) *P. shurbergia*, (g) *P. yunnanensis*, and (h) Pinewood nematode. Refer to M&M for data sources.

For host species, we selected seven *Pinus* species, *P. massoniana*, *P. taiwanensis*, *P. yunnanensis*, *P. armandii*, *P. bungeana*, *P. shunbergia*, and *P. tabuliformis*, as the focus of the study. The distributions of these species, *M. saltuarius* and the PWD were constructed from a number of online databases, including GBIF (https://www.gbif.org/), Chinese Virtual Herbarium (https://www.cvh.ac.cn/), and China National Knowledge Infrastructure (www.cnki.net). Google Earth (https://earth.google.com/web/) was used to query the longitude and latitude information corresponding to township names. Duplicate records and records of unknown coordinates were removed. The resultant records were then projected onto a map using ArcGIS (version 10.7, ESRI, USA). All distribution records were stored in the CSV format suitable for input into the MaxEnt model. Figure 2 presents the geographic distribution of the seven pine species and the PWN.

### 2.2 Bioclimatic variables

A total of 19 bioclimatic variables was initially selected for distribution modeling of the seven pine species and PWN (Table S1). Data for the bioclimatic variables were downloaded from the WorldClim database (http://www.worldclim.org). The data have a resolution of 30 seconds (1 km × 1 km), including four Shared Socioeconomic Pathways (SSPs: 126, 245, 370, and 585) (Hurtt et al., 2020). These pathways are designated as SSP1, SSP2, SSP3 and SSP5, representing low challenges for adaptation and mitigation, moderate challenges for adaptation and mitigation, high challenges for adaptation and mitigation, and high challenges for mitigation and low challenges for adaptation, respectively.

The data were converted to raster format and exported into ArcGIS. The clipping tool was used to extract data from within the rectangular area encompassing the study region. The projection tool was used to transform the coordinate system of the bioclimatic variables, unifying them under GCS‐WGS‐1984. Using China's administrative division map as a template, data from different bioclimatic variables were extracted from the global climate range. Pearson correlation analysis was used to screen the 19 bioclimatic variables (Benesty et al., 2009). Only variables with correlation coefficients |*r*| ≤ .8 were retained (Figure S1). The selected bioclimatic variables (Table S2) were used to model the distribution of the seven pine species and PWN.

### 2.3 MaxEnt model and ENMTools

We used the Maximum Entropy (MaxEnt, version 3.4.4) to model habitat suitability and the ENMTools to model niche overlap for the seven pine species, *M. saltuarius* and PWN. MaxEnt is a widely used machine learning algorithm to model species distribution based on presence‐only data and bioclimatic variables (Zhang et al., 2019). The basic response function of MaxEnt is a generalized linear model, given in Equation (1).

(1) $$H\left(\alpha \right)=\sum_{i=1}^{n}\;P_{1}\log\frac{1}{P_{i}}=-\sum_{i=1}^{n}\;P_{i}\log P_{i}$$

Under the premise of including known information, redundant information is eliminated when the entropy value is maximal. The model assumes that the random variable *α* contains *A*_1_, *A*_2_, *A*_3_, …, *A*_*n*_, a total of *n* possible results, where *p*_1_, *p*_2_, *p*_3_, …, *p*_*n*_ is the probability of each occurrence (Elith et al., 2011).

We utilized the Kuenm package (https://github.com/marlonecobos/kuenm) to optimize the regularization multiplier and feature class parameters within R version 4.2.1 (https://www.r‐project.org/) software (Cobos et al., 2019). These two parameters are crucial for constructing the species distribution model using MaxEnt version 3.4.4 (https://biodiversityinformatics.amnh.org/open_source/maxent/) software. During modeling, 75% of the data served as the training set. We then evaluated 1240 candidate models, encompassing combinations of 40 regularization multiplier settings (ranging from 0.1 to 4 at intervals of 0.1) and 29 feature class combinations. Model selection was based on statistical significance (partial receiver operating characteristic curve [ROC]), predictive ability (low omission rates), and complexity, in that order. Candidate models were screened to retain those demonstrating statistical significance. Subsequently, the model set was refined omitting variables contributing <5% of variance wherever possible. The model exhibiting the lowest delta Akaike's Information Criterion values (<2) was chosen among the significant and low‐omission candidate models (Figure S2).

The model utilized the ROC and the area under the ROC curve (AUC) to assess model accuracy. AUC values range from 0 to 1. The model's performance was classified as failure (AUC = 0.5–0.6), poor (AUC = 0.6–0.7), fair (AUC = 0.7–0.8), good (AUC = 0.8–0.9), and excellent (AUC > 0.9) (Elith et al., 2006). One advantage of this approach is that it provides a single measure of model performance, independent of any specific threshold selection. The higher the AUC value for given bioclimatic variables, the closer the correlation between the variables and the target species' geographic distribution model, and the better its predictive performance (Phillips et al., 2006; Wang et al., 2018).

The ROC analysis method was used to evaluate the simulation accuracy of the model and to identify the importance and optimal values of variables (Jiménez‐Valverde, 2012). The selected bioclimatic variables and occurrence data for the seven pine species and PWN were converted to ASCII format and uploaded to MaxEnt for distribution modeling. Here, 75% of the occurrence records were used to train the model, while the remaining 25% were used to test the model's predictive ability. The analysis was run for 10 repetitions with default settings for other parameters. The importance value of each bioclimatic variable was estimated by the Jackknife method.

We assessed habitat suitability based on the relationships between the present distribution of the target species and the bioclimatic variables. The predictive result outputs by MaxEnt software are Raster layers in ASCII format. For each species, the model calculated a habitat suitability index. It ranges 0–1, and ArcGIS software was used to visually express and reclassify the results. The index was then used to predict the potential distribution of the species under various scenarios of climate change.

The format conversion tool of ArcGIS was used to convert the data into Raster format so that they can be displayed in ArcGIS. The extract analysis was used to get the existence probability distribution map for each species. The probability values range 0–1. Based on this probability, an area was classified either as not suitable for PWN (0–.10), weakly suitable (.11–.25), moderately suitable (.26–.50), or highly suitable (.51–1). The criteria for determining habitat suitability levels are experience‐based (Liu et al., 2013, 2016).

We used ENMTools v1.3 (http://purl.oclc.org/enmtools) to determine the niche overlap between potential distributions of the seven pine species and PWN. ENMTools uses the known distribution data for a given species and related bioclimatic variables to calculate the ecological needs of the species. These needs were then projected into different spaces and times to construct the potential distribution of the species (Araújo & Peterson, 2012). Niche overlaps between the seven pine species and PWN were then calculated based on the Schoener *D* method, given in Equation (2).

(2) $$O_{\mathrm{ij}}=1-0.5\sum_{1}^{n}\;\left|P_{\mathrm{ia}}-P_{\mathrm{ja}}\right|$$

where $O_{\mathrm{ij}}$ represents the degree of niche overlap between species *i* and *j*; $P_{\mathrm{ia}}$ and $P_{\mathrm{ja}}$ represent the number of individuals using resource *a* (*a* = 1, 2, …, *n*) by species *i* and *j*, respectively. Schoener's *D* and Hellinger's *I* values were used to indicate the degree of niche overlap over the range 0–1; the larger the value, the higher the degree of niche overlap (Warren et al., 2010).

### 2.4 CLIMEX model

We used the CLIMEX model to predict potentially suitable habitats of the PWN vector, *M. alternatus*. It simulates species distribution by using environmental variables such as temperature, precipitation, and altitude, and biological factors such as competition and reproduction (Kriticos et al., 2012). CLIMEX (version 4) is an ecological modeling software and has been used to predict the geographic distribution and ecological niche of plant and animal species (Byeon et al., 2018). It can predict species' responses to climate change and assess risks to species distribution and ecological niche (Taylor et al., 2012) and has been widely used in many fields, including ecology, agriculture, forestry, and epidemiology (Kriticos et al., 2015). The output of CLIMEX is a quantitative representation of climate suitability for a given species and specified area via the ecoclimatic index (EI). EI was calculated using the annual growth index (GI_A_), annual stress index (SI), and stress interaction index (SX), given in Equation (3).

(3) $$\mathrm{EI}=GI_{A}\times \mathrm{SI}\times \mathrm{SX}$$

where GI_A_ represents the population growth potential during suitable seasons; SI represents the stress on populations during unsuitable seasons; and SX represents the interaction between these stress factors (Sutherst, 1999).

Ecoclimatic index defines a species' tolerance of particular climates and ranges between 0 and 100. The lower the EI, the less suitable climatic conditions are for a species' long‐term survival and vice versa. When EI is 0, it indicates harsh climatic conditions where a species cannot survive in the area for the long term, while an EI of 100 represents optimal conditions for species' survival. It is generally considered that when EI is <10, the species cannot survive in the area for the long term; EI > 10 indicates that the species can survive in the area for the long term; EI > 30 indicates that the climate conditions in the area are highly suitable for the species' long‐term survival (Kriticos et al., 2015). The parameters of the model are presented in Table S3.

CLIMEX assesses a species' potential range by considering a set of limiting factors, rather than relying solely on known distribution records (Bellard et al., 2018; Kriticos et al., 2003; Stephens et al., 2007; Webber et al., 2011). We used the CLIMEX model developed by Song and Xu (2006) to evaluate the distribution and dispersal ability of the PWN vector, *M. alternatus*.

To evaluate the potential risk areas for PWN in China, we constructed an integrated map of the centroid displacement of PWN and potential suitable areas for *Monochamus* species and the overlapping maps of the two under current and future climate conditions in 2050 and 2070. The potential distribution of the seven pine species and PWN predicted by MaxEnt was imported into ENMTools to calculate niche overlap. We used ArcGIS to calculate the sizes of suitable habitats and overlapping areas between the PWN, its vector, and the host species. We used the year 2070 as the end point of our study because changes in suitable habitat for PWN are stabilized at this time point.

## 3 RESULTS

### 3.1 Accuracy of the MaxEnt models and the contribution of each variable to overall predictions

Area under the ROC curve values of the runs ranged from 0.879 to 0.996 for the seven pine species, *M. saltuarius* and PWN (Table 1), indicating that the predictive values of the optimized MaxEnt model had good to excellent accuracy. Importance and contribution of bioclimatic variables to the potential distribution of the seven pine species and PWN are given in Table 2. The Jackknife method showed that variables related to precipitation (Bio12, 16, and 18) and temperature (Bio3, and 4) were the most important contributing factors to the potential distribution and the seven pine species, *M. saltuarius* and PWN.

**TABLE 1:** Results of MaxEnt model analysis.

Species	Default minus mean AUC values	Optimization minus mean AUC values
*Pinus massoniana*	0.965	0.965
*P. taiwanensis*	0.978	0.979
*P. armandii*	0.966	0.969
*P. bungeana*	0.980	0.990
*P. tabuliformis*	0.969	0.969
*P. shurbergia*	0.957	0.985
*P. yunnanensis*	0.984	0.986
*Monochamus saltuarius*	0.965	0.996
Pinewood nematode	0.877	0.879

*Note*: Values are the difference in the performance of the MaxEnt model under default and optimized settings. Mean AUC values are for the seven pine species, *M. saltuarius* and pinewood nematode.

Abbreviation: AUC, area under the receiver operating characteristic curve.

**TABLE 2:** Importance and contribution of bioclimatic variables to the potential distribution of the seven pine species, *Monochamus saltuarius* and pinewood nematode from the MaxEnt analysis.

Species	Variable	Contribution (%)	Permutation importance
*Pinus massoniana*	Bio12	51.9	25.3
	Bio3	24.1	41.1
*P. taiwanensis*	Bio18	65.5	78.7
	Bio3	17.3	12.3
*P. armandii*	Bio18	51.8	17.8
	Bio15	13.8	18.6
*P. bungeana*	Bio16	40.4	42.5
	Bio4	24.6	5.5
*P. tabuliformis*	Bio16	34.2	3.1
	Bio11	18.3	8.1
*P. shurbergia*	Bio16	50.0	56.5
	Bio4	19.8	0.7
*P. yunnanensis*	Bio18	56.9	33.8
	Bio4	16.8	9.0
*M. saltuarius*	Bio4	52.8	7.6
	Bio18	40.9	30.1
Pinewood nematode	Bio18	74.4	54.3
	Bio3	15.9	24.9

### 3.2 Potential distribution of pine species and PWN

**FIGURE 3:**
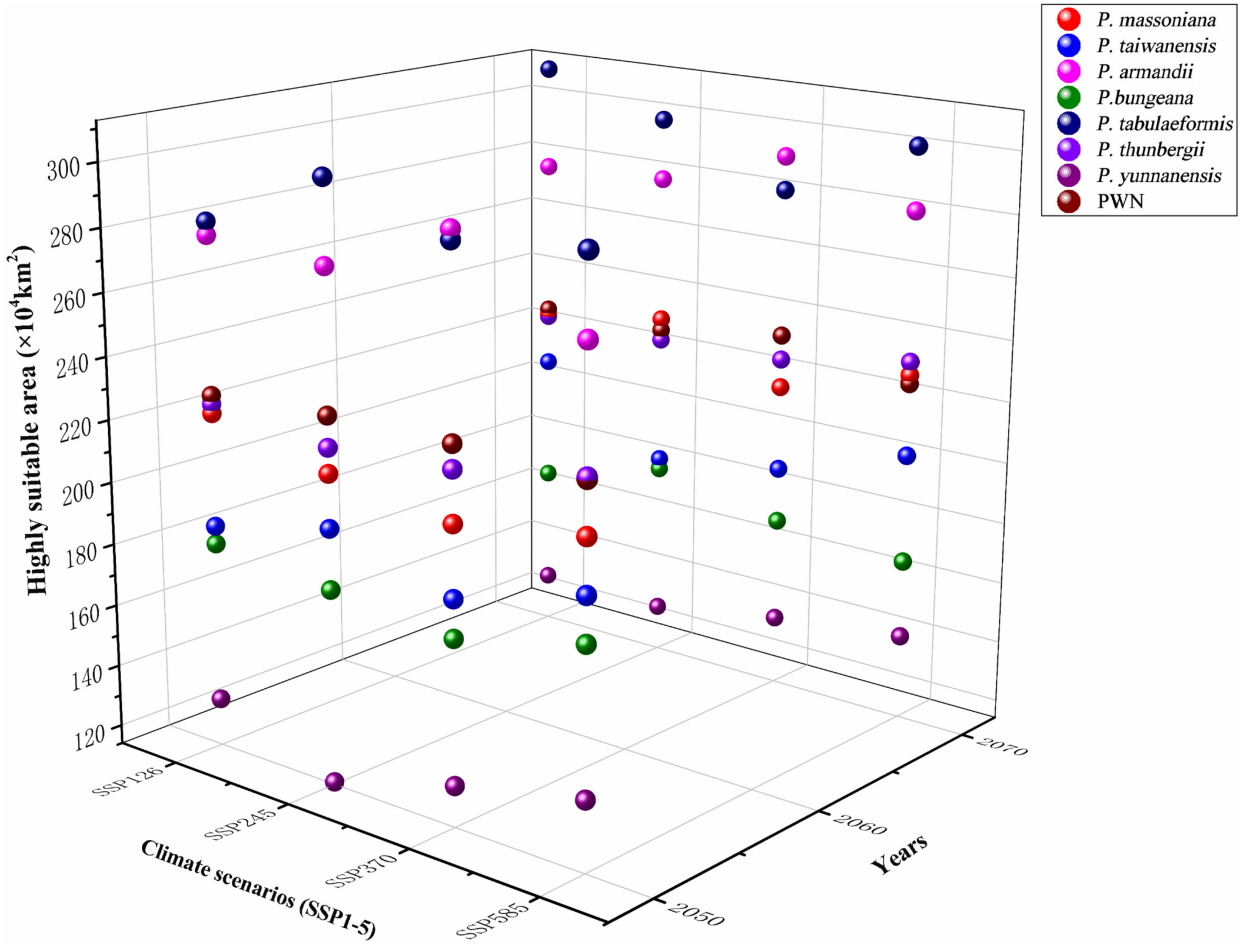
Three‐dimensional graph showing the percentage changes in the area of highly suitable habitat for the seven pine species and the pinewood nematode in 2050 and 2070 and under four climate scenarios (SSP126, SSP245, SSP370 and SSP585). SSP, Shared Socioeconomic Pathway.

**FIGURE 4:**
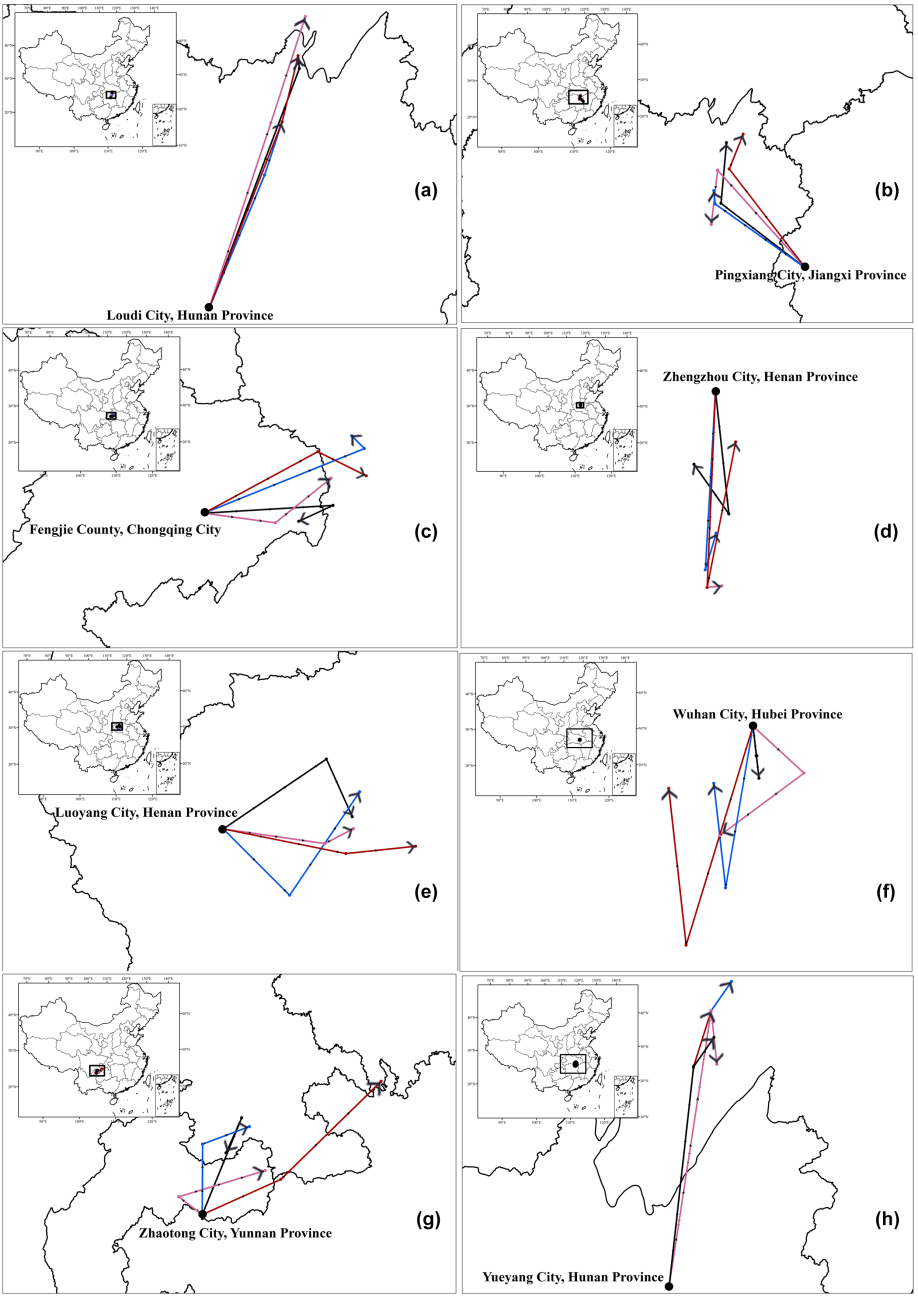
The geometric centroids of potential suitable habitats for pinewood nematode and the seven pine species in 2050 to 2070 under four climate change scenarios. (a) Pinewood nematode, (b) *Pinus massoniana*, (c) *P. taiwanensis*, (d) *P. armandii*, (e) *P. bungeana*, (f) *P. tabulaeformis*, (g) *P. shurbergia*, (h) *P. yunnanensis*. The black, pink, blue, and red lines represent SSP126, 245, 370, and 585 outputs, respectively. SSP, Shared Socioeconomic Pathway.

Under present climatic conditions, the area suitable for PWN is 4.4 (*P. armandii*), 3.0 (*P. bungeana*), 3.2 (*P. massoniana*), 5.3 (*P. tabuliformis*), 2.8 (*P. taiwanensis*), 3.1 (*P. thunbergia*) and 3.4 (*P. yunnanensis*) million km^2^ (mkm^2^) (Figure S3II–VIII). Niche ranges of the seven pine species will expand under various scenarios of climate change in 2050 and 2070 by 7.3% to 43.6% (Figure 3; Figure S3II–VIII). The area suitable in 2050 and 2070 will increase by 30.5% and 27.1% under SSP245, by 21.2% and 28.0% under SSP370, and by 35.4% and 25.3% under SSP370, respectively. The area of high suitability is projected to gradually move from central China to northern China (Figure S2I; Figure 4a).

Shifts in the geographic centroids varied for the seven pine species and PWN in 2050 and 2070 under various scenarios (Figure 4). For PWN, *P. massoniana*, *P. taiwanensis*, and *P. yunnanensis*, the centroids will shift northward (Figure 4a–c,h); for *P. bungeana*, the centroid will shift southward (Figure 4e); for *P. armandii*, the centroid will shift eastward (Figure 4d). In contrast, the centroid of *P. shurbergia* and *P. tabuliformis* will shift southward under SSP126 and SSP245 but will shift northwestward under SSP370 and SSP585 (Figure 4f,g).

### 3.3 Potential distribution of *Monochamus* species and PWN

**FIGURE 5:**
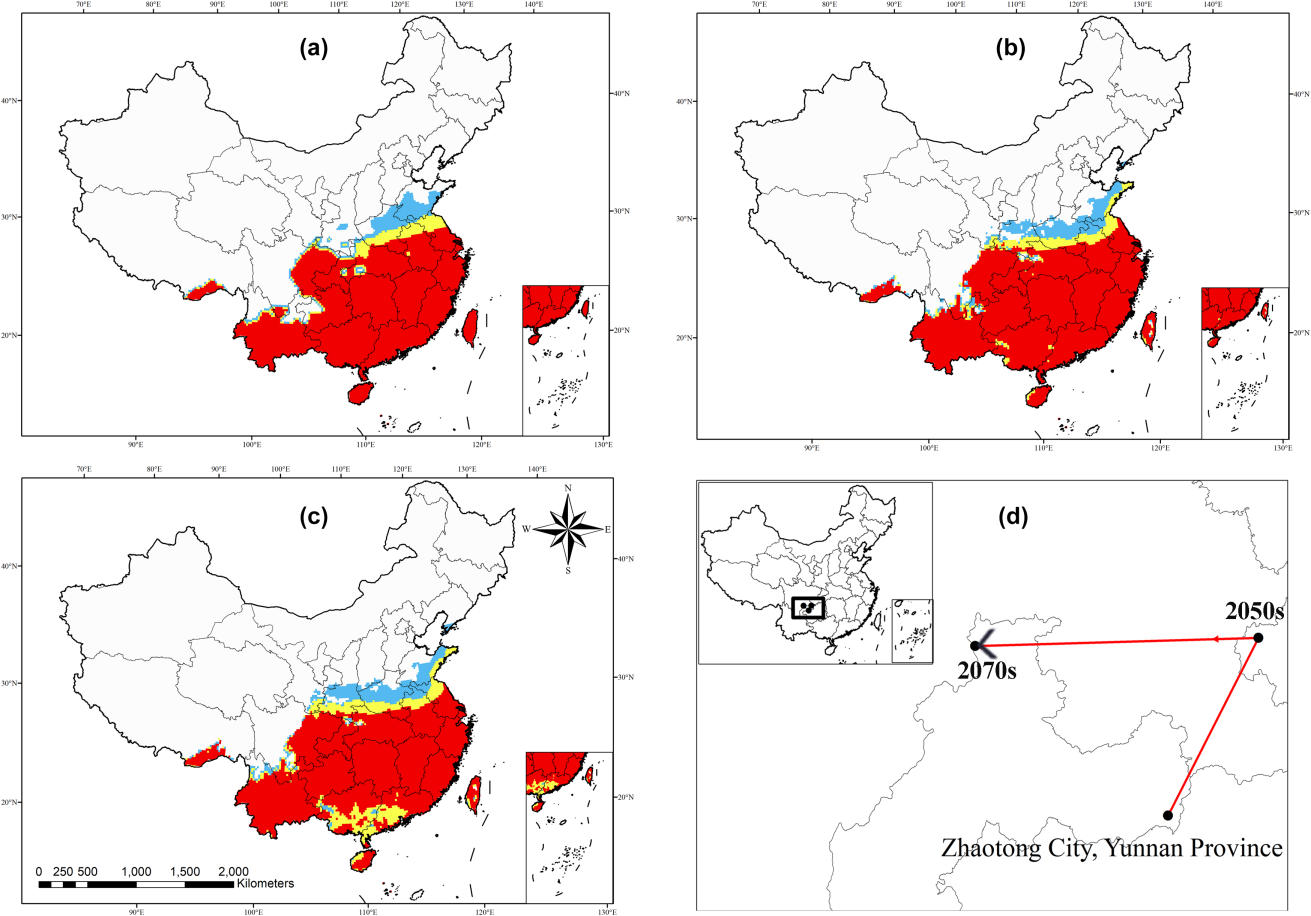
Spatial distribution of potentially suitable areas for the *Monochamus alternatus* in China, based on the CLIMEX model analysis. (a) current habitats; (b) potential habitats in 2050; (c) potential habitats in 2070. Low‐suitability areas (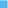): 10 < EI < 20; moderate‐suitability areas (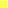): 20 < EI < 30; high‐suitability areas (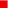): 30 < EI < 100. (d) geometric centroids of potential suitable habitats for the *M. alternatus*. EI, ecoclimatic index.

The PWN vector, *M. alternatus*, is distributed mainly in subtropical and tropical China, including the eastern part of Sichuan, parts of Yunnan and Guizhou, and Hubei. Some parts of temperate China such as Henan, Shandong and Shaanxi were classified as low suitability zones of the vector (Figure 5). *M. saltuarius* is distributed mainly in northeastern China, including Heilongjiang, Jilin, Liaoning and north‐eastern Inner Mongolia and Shanxi, Shandong, Hebei, Beijing, Tianjin (Figure S4).

The potentially suitable area for *M. alternatus* and *M. saltuarius* is 3.7 mkm^2^, accounting for 38.5% of the total area of China. Among this area, the high‐suitability area is 2.1 mkm^2^, accounting for 21.9% of China. The moderately suitable area is 1.23 mkm^2^, whereas the low suitability area is 0.95 mkm^2^. In 2050, the potential suitable area for these vectors will reach 4.6 mkm^2^, an increase of 24.3% compared with the current distribution. The high‐suitability area will reach 2.6 mkm^2^, or 27.1% of China, an increase of 23.8%; the moderately suitable area will reach 1.11 mkm^2^; and the low‐suitability area will reach 0.87 mkm^2^. In 2070, the potential suitable area of the vector will reach 4.62 mkm^2^, an increase of 24.9% over the present distribution area. The high suitability area will reach 2.4 mkm^2^, accounting for 25% of China, an increase of 0.4% and a decrease of 2.1% from the 2050 distribution area; the moderately suitable area will reach 1.22 mkm^2^, and the low suitability area will reach 0.88 mkm^2^.

The potential distribution area of *Monochamus* species will further expand between 2050 and 2070, with high suitability areas covering the southern regions of China and gradually extending towards the northern regions (Figure 5; Figure S4).

### 3.4 Niche overlap between host species and PWN

Table 3 shows the niche overlap between the seven pine species and PWN. Among the seven pine species, niche overlap was highest for *P. massoniana* (*D* = 0.77, *I* = 0.93), *P. taiwanensis* (*D* = 0.76, *I* = 0.94), and *P. shurbergia* (*D* = 0.75, *I* = 0.93).

**TABLE 3:** Overlap of potential habitat areas of the seven pine species and pinewood nematode (PWN).

*D* (above the diagonal)/*I* (below the diagonal)^a^	PWN	PM	PT1	PY	PA	PB	PT2	PS
PWN	1	0.77	0.76	0.53	0.64	0.54	0.55	0.75
PM	0.93	1	0.74	0.55	0.66	0.60	0.57	0.78
PT1	0.94	0.91	1	0.52	0.59	0.47	0.47	0.71
PY	0.80	0.81	0.80	1	0.67	0.40	0.51	0.49
PA	0.86	0.90	0.86	0.91	1	0.59	0.74	0.60
PB	0.80	0.87	0.76	0.71	0.84	1	0.63	0.68
PT2	0.79	0.86	0.75	0.80	0.93	0.88	1	0.58
PS	0.93	0.96	0.91	0.78	0.86	0.91	0.84	1

Abbreviations: PA, *Pinus armandii*; PB, *P. bungeana*; PM, *P. massoniana*; PS, *P*. shurbergia; PT1, *P. taiwanensis*; PT2, *P. tabuliformis*; PY, *P. yunnanensis*.

^a^ Schoener's *D* and Hellinger's *I* values indicate the degree of niche overlap, and range 0–1, such that the larger the value, the higher the degree of niche overlap.

### 3.5 Results for ensemble models (CLIMEX and MaxEnt) in evaluating potential risk areas of PWN

**FIGURE 6:**
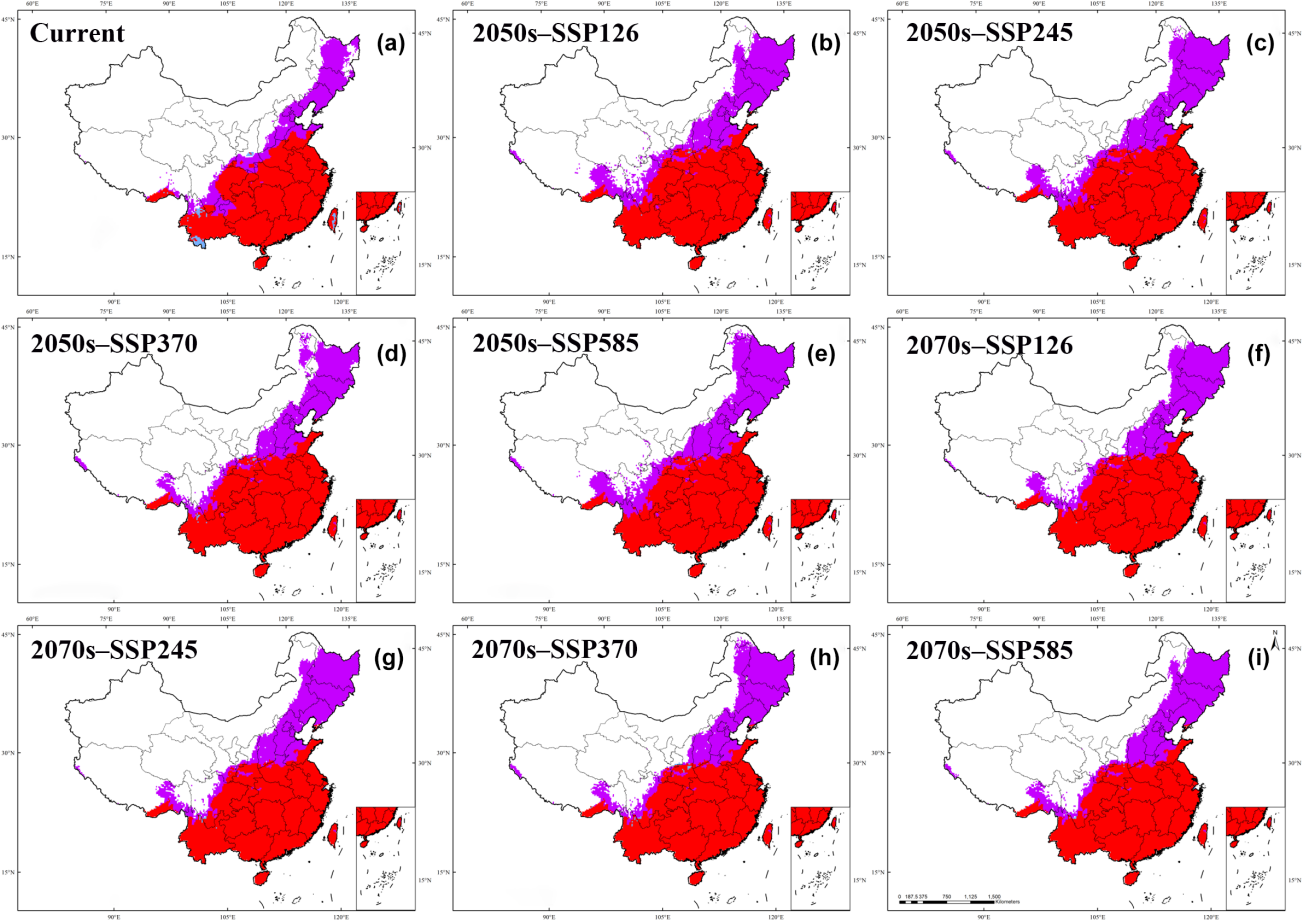
Overlap areas of pinewood nematode (PWN) and *Monochamus saltuarius*, based on the MaxEnt model. (a) Current; (b) S1‐50; (c) S2‐50; (d) S3‐50; (e) S5‐50; (f) S1‐70; (g) S2‐70; (h) S3‐70; (i) S5‐70. S1, scenario SSP126; S2, scenario SSP245; S3, scenario SSP370; S5, scenario SSP585; S1‐50: S1 in 2050; S2‐50: S2 in 2050; S3‐50: S3 in 2050; S5‐50: S5 in 2050; S1‐70: S1 in 2070; S2‐70: S2 in 2070; S3‐70: S3 in 2070; S5‐70: S5 in 2070. Suitable areas of PWN (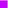); the suitable areas for PWN + *M. alternatus* (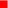).

The distribution of PWN, represented by the overlap of the nematode and *M. alternatus*, will spread to the northern regions of China, including parts of Gansu, Shaanxi, Henan, Shandong, Shanxi and Hebei provinces (Figure S5). Severe outbreaks of PWN will occur in eastern Sichuan, southern Henan, Chongqing, Guizhou, Hubei, Hunan, Anhui, Jiangsu, Shanghai, Jiangxi, Zhejiang, Guangxi Zhuang Autonomous Region, Guangdong, and Fujian. In contrast, the western and far northern regions, including Xinjiang Uyghur Autonomous Region, Qinghai, Ningxia Hui Autonomous Region, and Inner Mongolia will remain at low risk of the disease (Figure 6).

## 4 DISCUSSION

We developed potential distribution models for PWD and its major participants: seven host species (pines), the parasite (PWN), and its beetle vector, under various climate change scenarios. While studies using single models to predict the spread of this disease have had some success (Ikegami & Jenkins, 2018; Ouyang et al., 2022; Yoshimura et al., 1999), recent studies have shown that the use of integrated models, such as CLIMEX and MaxEnt used here, can lead to more accurate predictions (Narouei‐Khandan et al., 2020; Yoon et al., 2023). In our study, these programs produced high AUC values (0.879–0.996) (Table 1), indicating that the model results have a high degree of accuracy and reliability in predicting the potential distribution of this disease.

From the present to the years, 2050 and 2070, the predicted areas suitable for the PWN and pine hosts will increase substantially under the four scenarios produced (SSP126, SSP245, SSP370 and SSP585) (Figure 3; Table S4). The only exceptions are *P. tabuliformis* and *P. yunanensis* and PWN. Between 2050 and 2070, the suitable habitat for these two pine species decreased by 1.5% to 3.4% under SSP126, SSP245, and SSP585. The suitable habitat for PWN decreased by 1.5% to 7.5% under SSP245 and SSP585. This decrease in suitable habitat of PWN is most likely related to the changes in precipitation of the warmest quarter (Bio18) under extreme conditions (Table 2; Table S1) from 2050 to 2070. Our predictions are based on the MaxEnt model and on data for PWD occurrence over 8 years (2015–2022) at the township level. As machine learning‐based algorithms, such as the MaxEnt model, are dependent on the accuracy of occurrence data, numerous occurrence records collected over a number of years will increase the reliability of the model and help improve its predictive value. In contrast, previous studies on PWD were often based on occurrence data for a single year or at broader scales (district level) (Matsuhashi et al., 2020; Ouyang et al., 2022; Robinet et al., 2009; Wang, Peng, et al., 2022; Wang, Wang, et al., 2022).

Temperature and precipitation are the most important climatic factors affecting the distribution of the vector. The areas most suitable are characterized by warm and humid climates, while areas with dry and cold climates are generally not suitable for this beetle. The temperature‐related variables contribute to the potential distribution of the disease through their influence on PWN and its vector. Low temperatures (<−3.2°C) limit the survival of nematodes, thus regulating the distribution of the disease. For a given area, an increase in precipitation will increase the probability and severity of the disease, as both the nematode and beetle are favored by the humid climate (Roques et al., 2015). As climate change continues to intensify in the future, the areas suitable for the vector will gradually expand northward (Figure 5). To control the northward expansion of the disease, measures should be taken to prevent the northward migration of this beetle. This prediction is consistent with the results of previous studies (Gao et al., 2019; Ouyang et al., 2022; Tang et al., 2021; Wang et al., 2023).

Interactions between temperature and precipitation may also influence the distribution of the disease by affecting the disease susceptibility of the pine species. Low precipitation (drought) and high temperatures can induce drought stress in the host species, rendering them more susceptible to nematode infection, thus increasing the risk of PWD (Roques et al., 2015). PWD was most severe in subtropical China in 2022 (https://www.forestry.gov.cn/), coinciding with the most severe drought over the last 100 years in the area (https://www.cma.gov.cn).

Our results show that the geographic centroids of PWN and three of the host species, *P. massoniana*, *P. taiwanensis*, and *P. yunnanensis*, will shift northward under various climate change scenarios (Figure 4). In addition, we analyzed the niche overlap between the seven pine species, the PWN and its beetle vector. The suitable niches of three pine species, *P. massoniana*, *P. taiwanensis* and *P. shurbergia*, overlap strongly with the PWN and beetle, implying that these three species are at high risk of PWD (Figure S6III,V,VI). The risk of PWD, represented by the overlap of the nematode and its vector, will spread to the northern regions of China, including Gansu, Shaanxi, Henan, Shandong, Shanxi and Hebei provinces. The disease will spread rapidly once the climate becomes favorable for the PWN and beetle. For instance, over the past half‐century, the average temperature in Shanxi Province has increased by 1.2°C, while annual precipitation has decreased by 99 mm (Fan & Wang, 2011). The combined effects of rising temperatures and decreasing precipitation in the northern region of China are two‐fold. First, they reduce the vigor of pine species, making them susceptible to the disease. Second, high temperatures increase the vigor of PWN and the beetle, further exacerbating the spread of the disease. The beetle, in particular, is highly sensitive to changes in temperature and precipitation (Gao, Liu, Zhao, & Cui, 2023; Panesar et al., 1994). It is most active in areas of warm (15–30°C) and humid (0.55–1.35 water vapor in kg/m^3^) climate. In contrast, its growth and development are limited in areas of dry and cold climates (An et al., 2019; Roques et al., 2015). Rising temperatures and lower humidity under climate change will render some of the northern regions suitable for the beetle, resulting in the gradual northward movement of PWD.

Using MaxEnt and CLIMEX models, our integrated modeling approach was able to predict the potential distribution of PWD, PWN, and its beetle vector. However, our modeling results are subject to several limitations. First, our model is based only on some key environmental factors—other factors such as soil type and land use, which may influence the distribution of this disease, were not considered. Second, our study only predicted the potential distributions of PWN and its vector in specific regions—their population ecology and evolutionary history were not considered. Third, our model was able to predict the overall distribution ranges of PWD and its three participants (nematode, vector, and host species), but it did not integrate the effects of local environmental conditions on their distribution. Further studies are needed to explore these limitations to make the prediction more comprehensive and accurate.

## 5 CONCLUSIONS

Our study demonstrated that the integrated modeling approach of MaxEnt‐CLIMEX is effective in assessing the potential risk of PWD in China under climate change. This approach can effectively predict the potential distribution of PWN, its beetle vector, and the seven pine species and areas at high risk of PWD under various climate change scenarios. Our models show that suitable habitat for PWN will expand by 21%–35% from its current area, in 2050 and 2070; suitable habitats for the beetle will expand by 8.5%–11.1%. As temperature rises and precipitation decreases, some northern areas will become potentially suitable habitats of the PWN and its vector. As a result, the geographic centroids of PWN and the beetle will gradually shift northward, resulting in the spread of PWD to the northern regions of China. In contrast, the western and far northern regions, including Xinjiang Uyghur Autonomous Region, Qinghai and Inner Mongolia will be at low risk of the disease.

## AUTHOR CONTRIBUTIONS

**Lin Chen:** Data curation (equal); formal analysis (equal); investigation (equal); methodology (equal); software (equal); validation (equal); visualization (equal); writing – original draft (equal); writing – review and editing (equal). **Wenxiong Lu:** Formal analysis (equal); investigation (equal); methodology (equal); validation (equal). **Byron B. Lamont:** Methodology (equal); software (equal); supervision (equal); writing – review and editing (equal). **Yu Liu:** Investigation (equal); methodology (equal); software (equal); validation (equal). **Pujie Wei:** Investigation (equal); methodology (equal); software (equal). **Weixing Xue:** Investigation (equal); methodology (equal). **Zixuan Xiong:** Investigation (equal); methodology (equal). **Li Tang:** Validation (equal). **Yongjian Wang:** Data curation (equal); formal analysis (equal); investigation (equal); project administration (equal); validation (equal). **Pengcheng Wang:** Conceptualization (equal); investigation (equal); project administration (equal); supervision (equal); validation (equal); writing – review and editing (equal). **Zhaogui Yan:** Conceptualization (equal); methodology (equal); resources (equal); supervision (equal); visualization (equal); writing – review and editing (equal).

## CONFLICT OF INTEREST STATEMENT

The authors declare that they have no known competing financial interests or personal relationships that could have appeared to influence the work reported in this paper.

## Supporting information


Data S1.

## Data Availability

All data are in the main text or the Supporting Information.
