# Supplementary material for: Modeling the distribution of pine wilt disease in China using the ensemble models MaxEnt and CLIMEX
Source: Ecol Evol. 2024 Sep 19;14(9):e70277. doi: 10.1002/ece3.70277 (PMC11412742; doi:10.1002/ece3.70277)
Supplement: Supplementary file 1 — Data S1. [file ECE3-14-e70277-s001.docx]

**Supplementary Information**


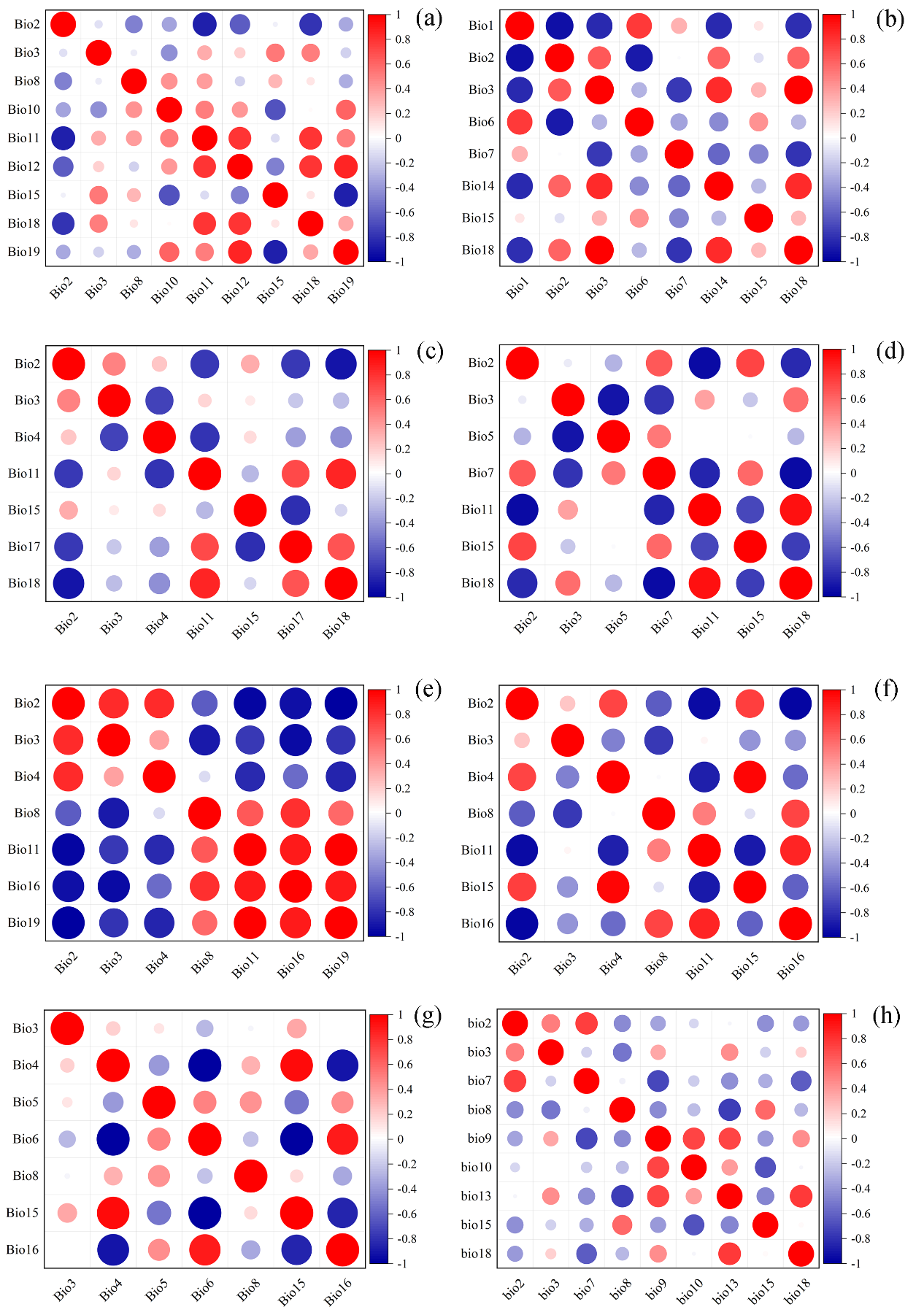


**Fig. S1** Pearson correlation values between the selected bioclimatic variables for the seven host species and pinewood nematode. **(a)** *Pinus massoniana*, **(b)** *P. taiwanensis,* **(c)** *P. yunnanensis*, **(d)** *P. armandii*, **(e)** *P. bungeana*, **(f)** *P. tabuliformis*, **(g)** *P. shurbergia*, **(h)** Pinewood nematode*.*


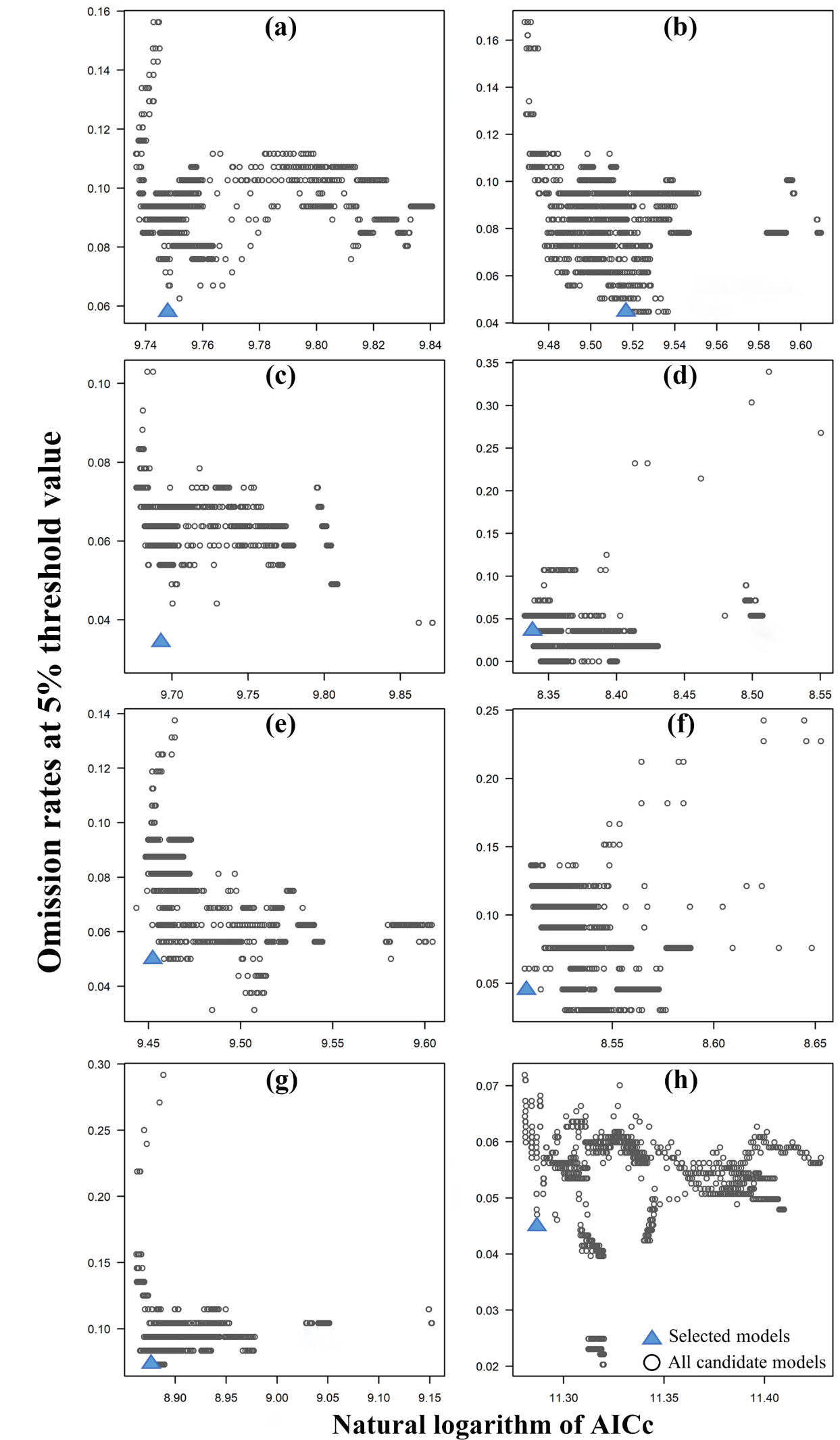


**Fig. S2** Omission rates and AICc values for all non-significant and selected ‘best’ candidate models for the seven pine species and pinewood nematode. Models were based on statistical significance, omission rates, and AICc criteria. **(a)** *P. massoniana*, **(b)** *P. taiwanensis*, **(c)** *P. armandii*, **(d)** *P. bungeana*, **(e)** *P. tabuliformis*, **(f)** *P. shurbergia*, **(g)** *P. yunnanensis*, and **(h)** Pinewood nematode.

**Fig. S3** Potential changes in habitat areas for pinewood nematode and the seven pine species under various climate scenarios in 2050 and 2070. **(a)** S1-50; **(b)** S2-50; **(c)** S3-50; **(d)** S5-50; **(e)** S1-70; **(f)** S2-70; **(g)** S3-70; **(h)** S5-70. S1, scenario SSP126; S2, scenario SSP245; S3, scenario SSP370; S5, scenario SSP585; S1-50: S1 in 2050; S2-50: S2 in 2050; S3-50: S3 in 2050; S5-50: S5 in 2050; S1-70: S1 in 2070; S2-70: S2 in 2070; S3-70: S3 in 2070; S5-70: S5 in 2070. Highly suitable (
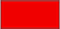
); moderately suitable (
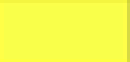
); weakly suitable (
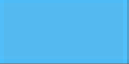
)

(I) Pinewood nematode


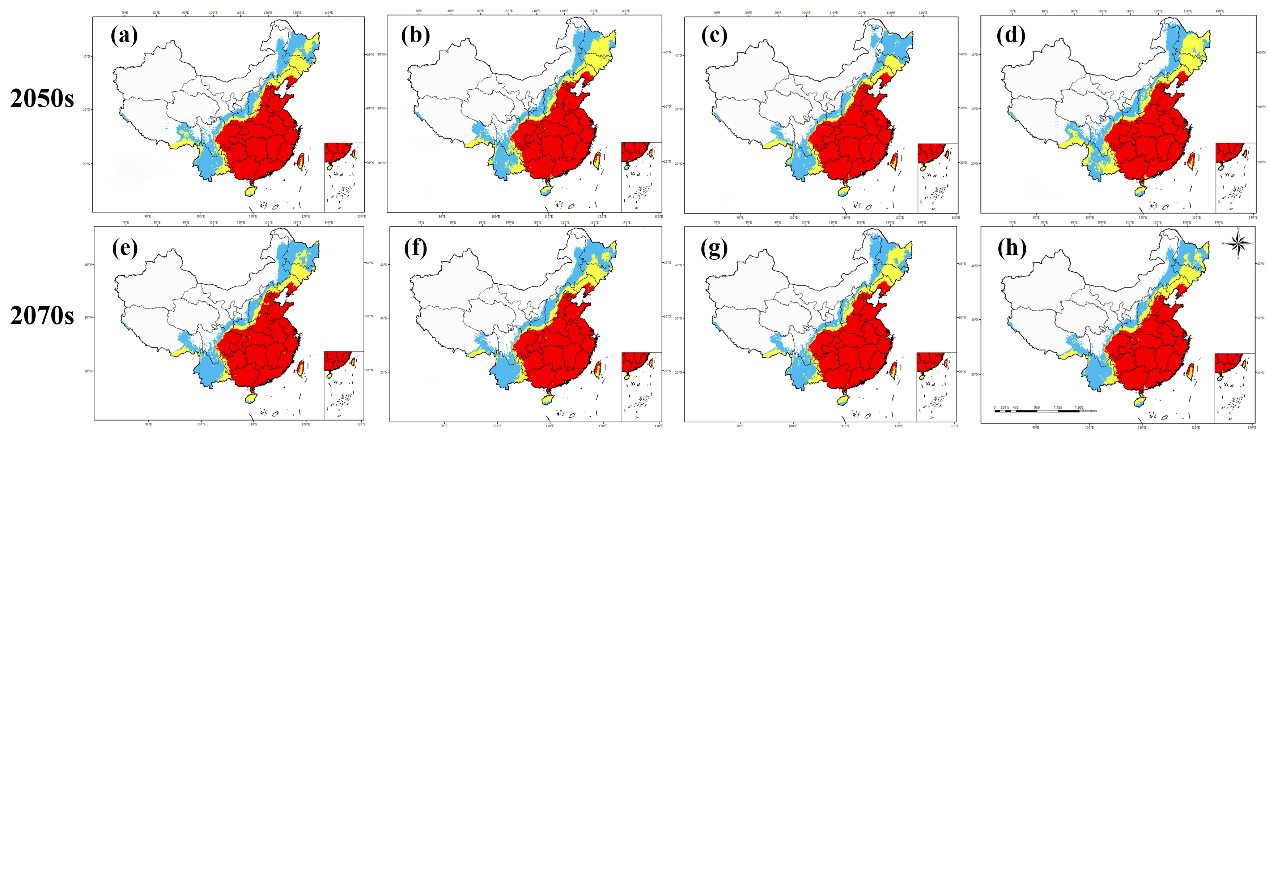


(II) *P. armandii*


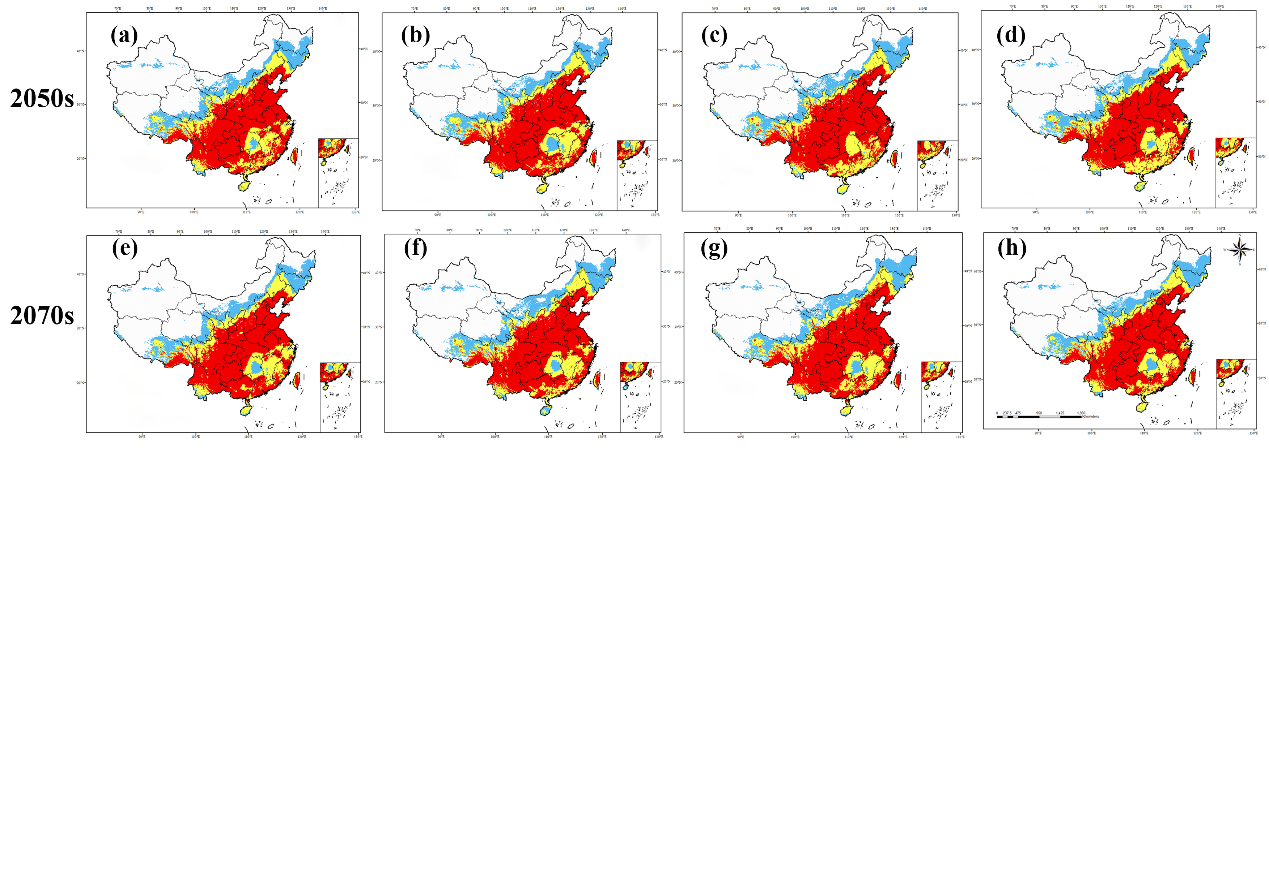


(III) *P. bungeana*


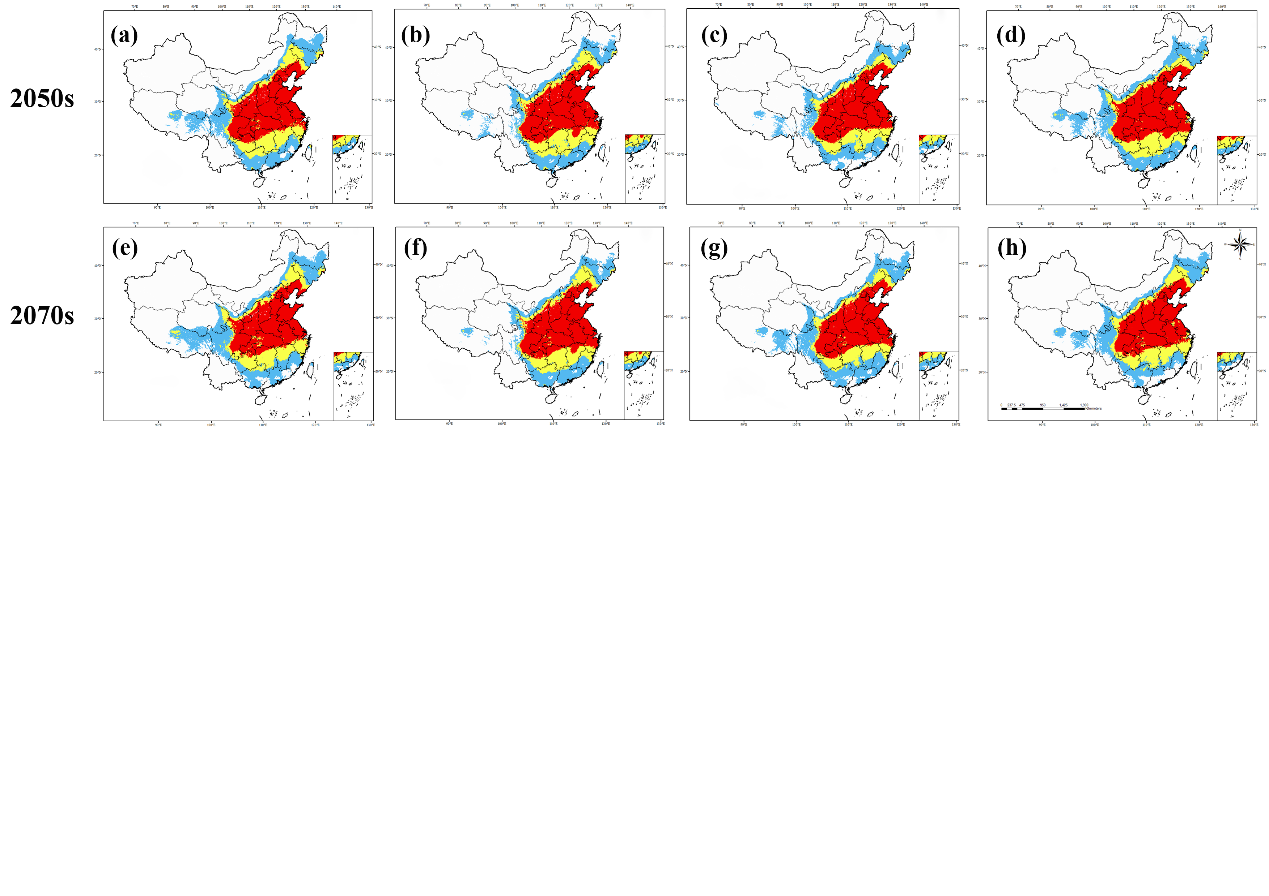


(IV) *P. massoniana*


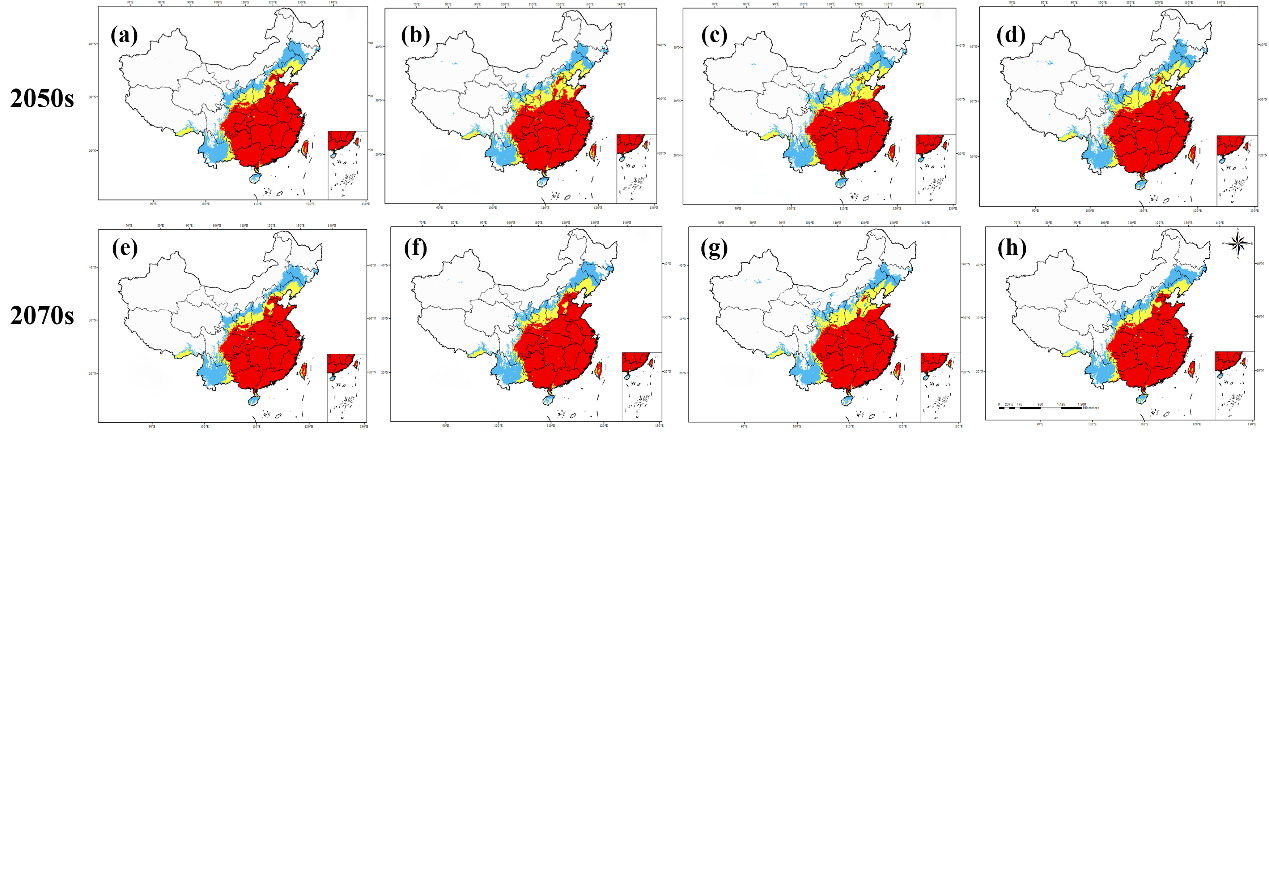


(V) *P. tabuliformis*


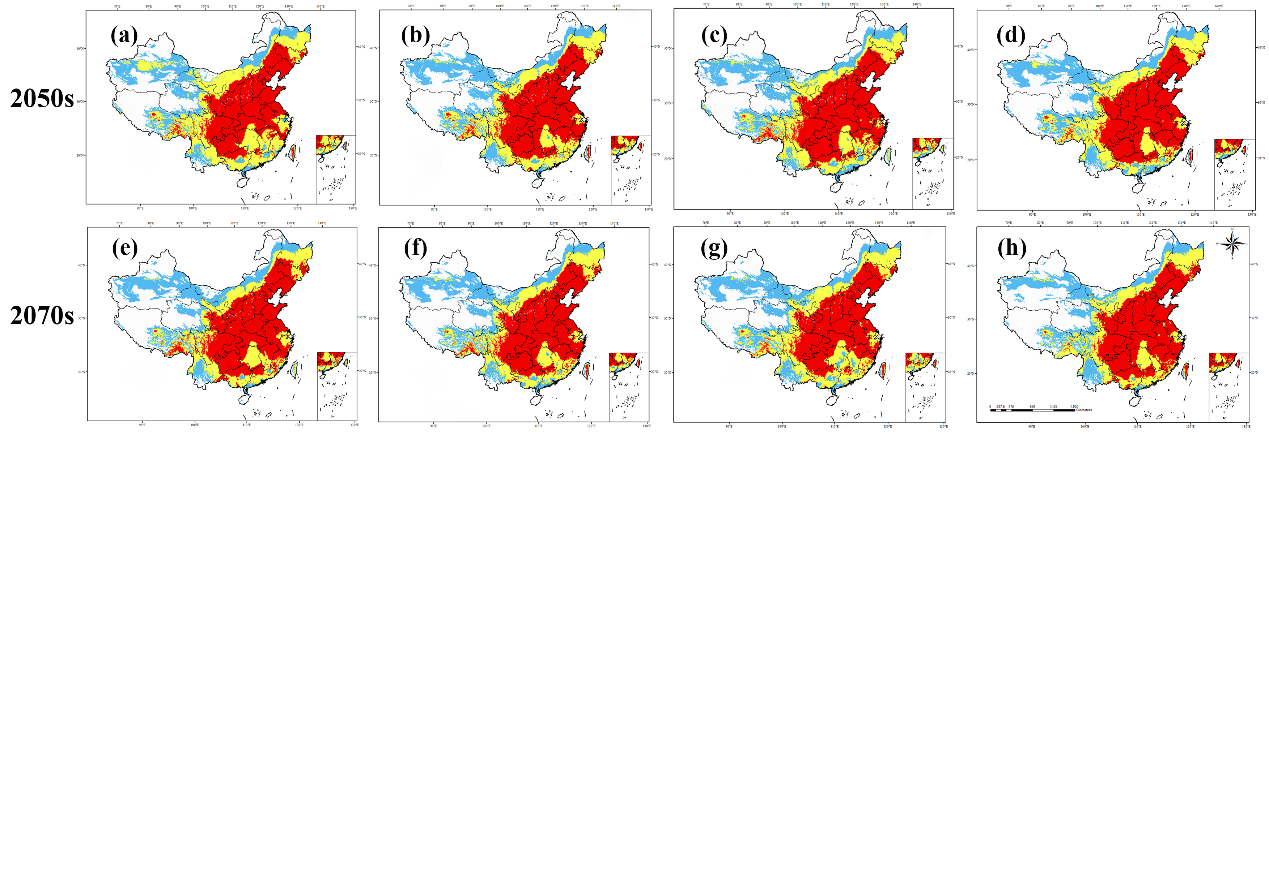


(VI) *P. taiwanensis*


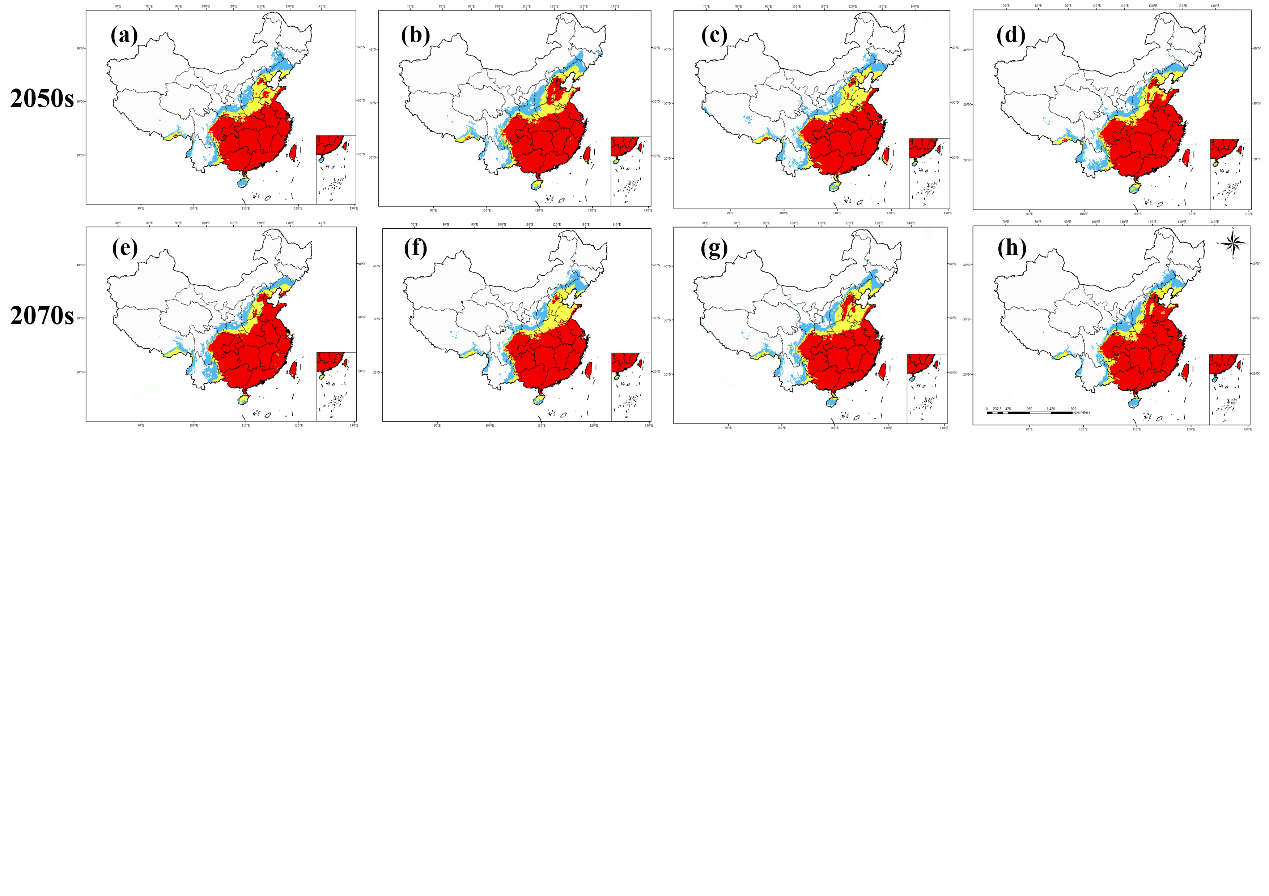


(VII) *P. shurbergia*


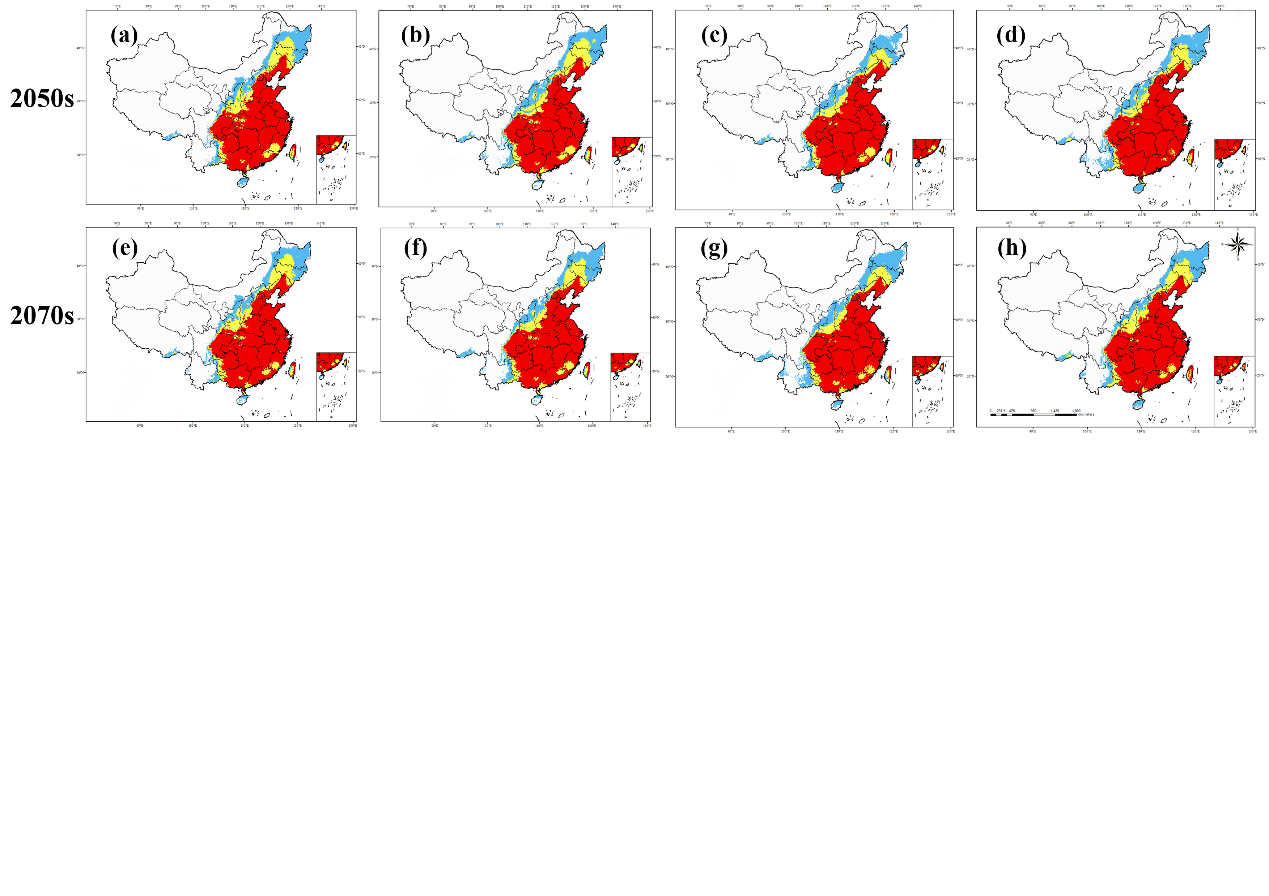


(VIII) *P. yunnanensis*


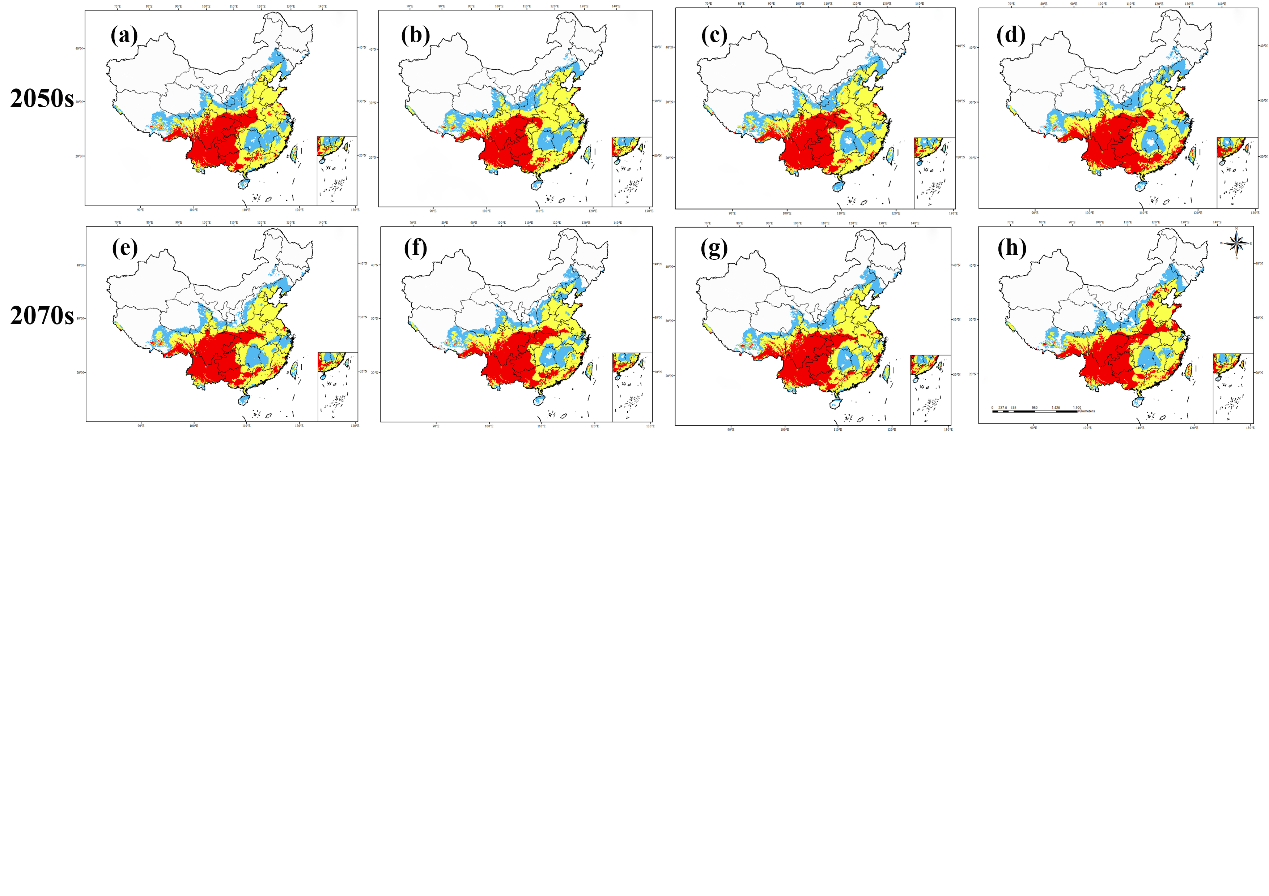


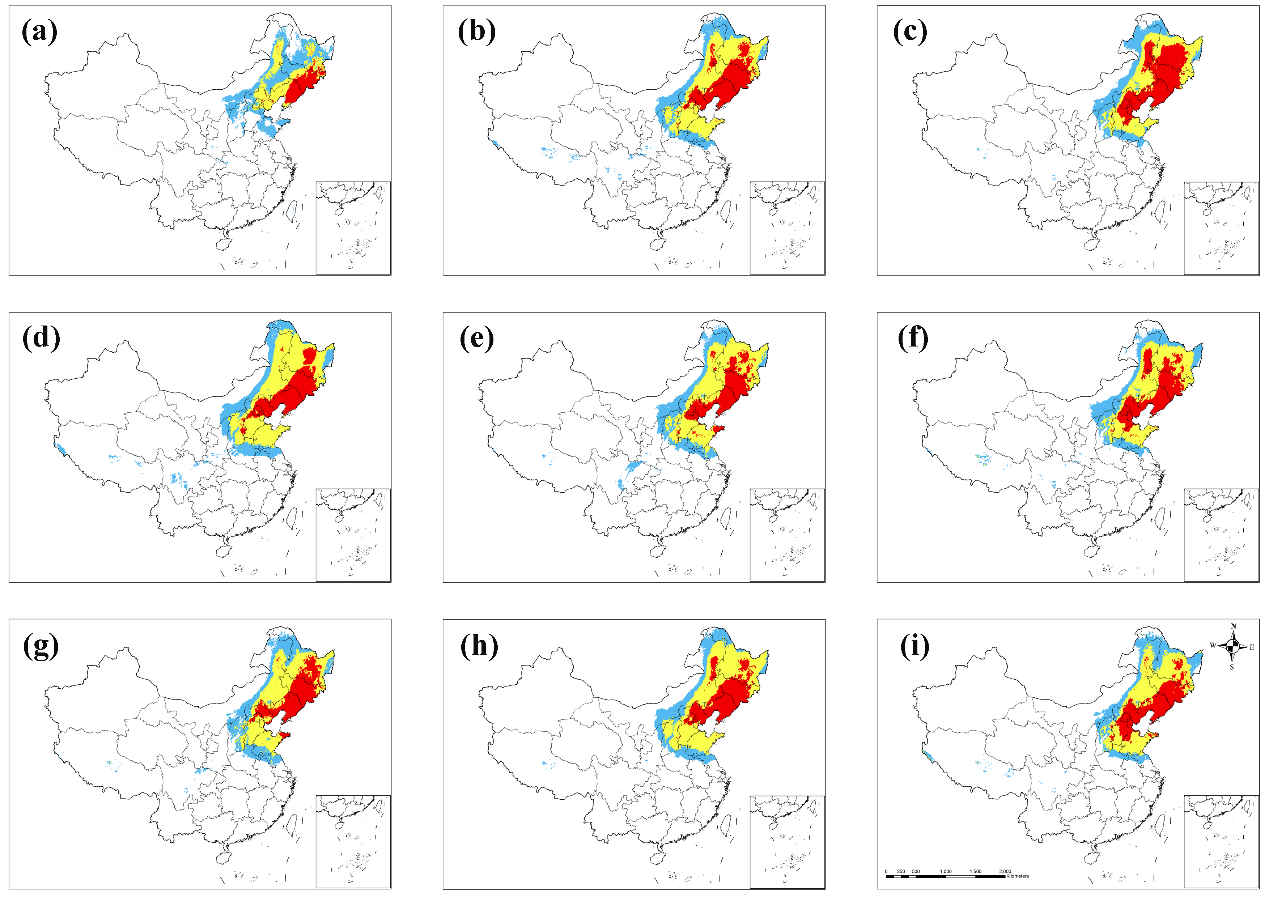


**Fig. S4** Potential changes in habitat areas for *Monochamus saltuarius* under various climate scenarios in 2050 and 2070. **(a)** Current; **(b)** S1-50; **(c)** S2-50; **(d)** S3-50; **(e)** S5-50; **(f)** S1-70; **(g)** S2-70; **(h)** S3-70; **(i)** S5-70. S1, scenario SSP126; S2, scenario SSP245; S3, scenario SSP370; S5, scenario SSP585; S1-50: S1 in 2050; S2-50: S2 in 2050; S3-50: S3 in 2050; S5-50: S5 in 2050; S1-70: S1 in 2070; S2-70: S2 in 2070; S3-70: S3 in 2070; S5-70: S5 in 2070. Highly suitable (
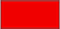
); moderately suitable (
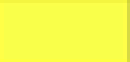
); weakly suitable (
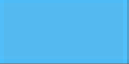
)


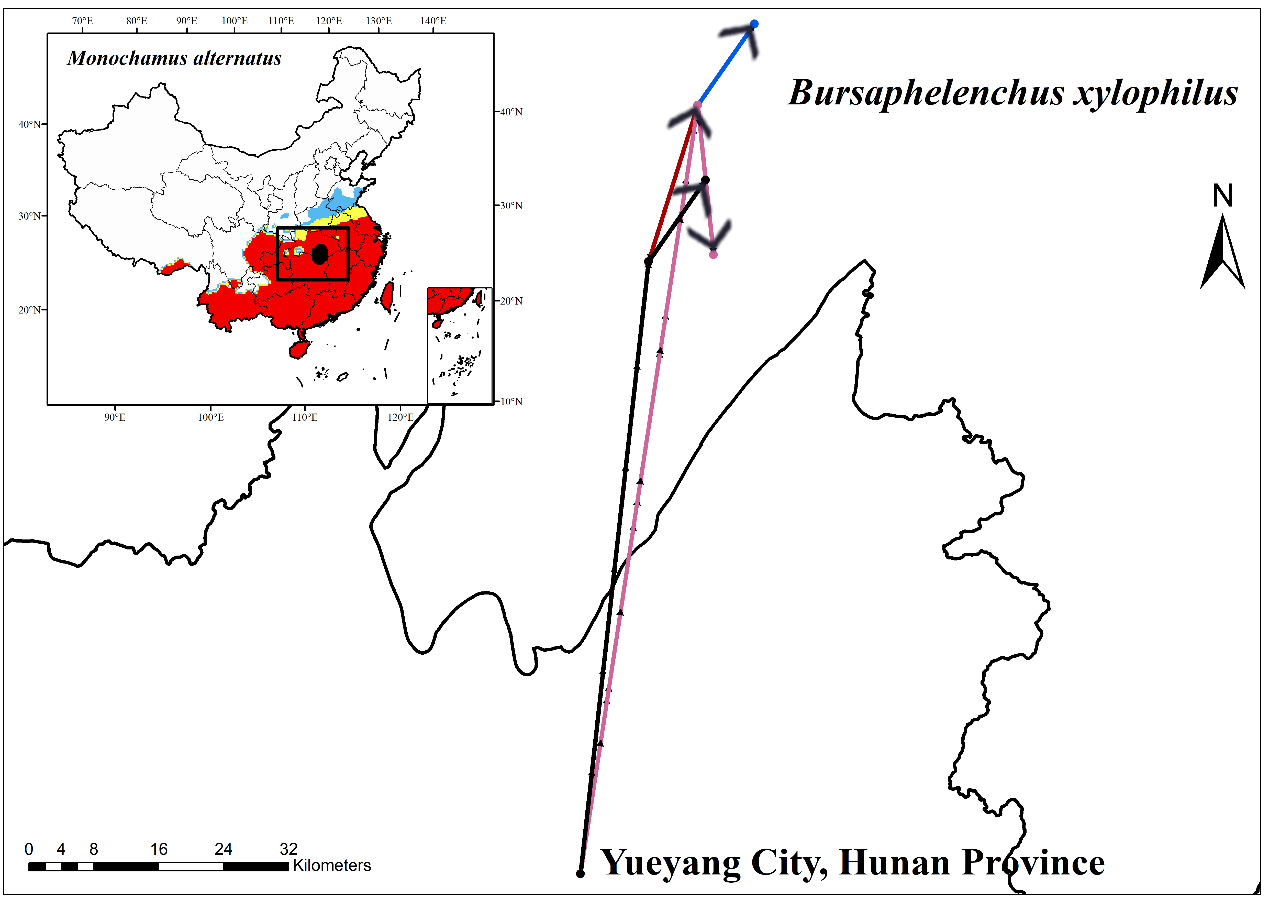


**Fig. S5** Spatial distribution of potential suitable habitat for pinewood nematode (*Bursaphelenchus xylophilus*) and *Monochamus alternatus* in China based on the CLIMEX model analysis. Potential suitable habitat for the insect vector was determined by the CLIMEX model. The geometric centroid shifts were determined in ArcGIS 10.7. The black, pink, blue, and red line represent SSP126, 245, 370, and 585, respectively. Highly suitable (
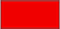
); moderately suitable (
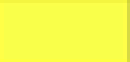
); weakly suitable (
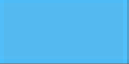
)

**Fig. S6** Overlap of suitable habitat of pinewood nematode, *Monochamus alternatus* and the seven pine species under various climate scenarios in current, 2050 and 2070. **(a)** Current; **(b)** S1-50; **(c)** S2-50; **(d)** S3-50; **(e)** S5-50; **(f)** S1-70; **(g)** S2-70; **(h)** S3-70; **(i)** S5-70. Refer Fig. S1 for abbreviation details. The suitable areas of PWN (
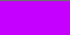
); *M. alternatus* (
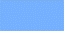
); PWN + *M. alternatus* (
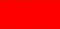
).

(I) *P. armandii*

Overlapping range of suitable areas of *P. armandii* (
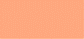
); PWN + *P. armandii* (
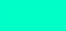
); *M. alternatus* + *P. armandii* (
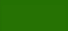
); *M. alternatus* + PWN + *P. armandii* (
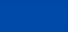
).


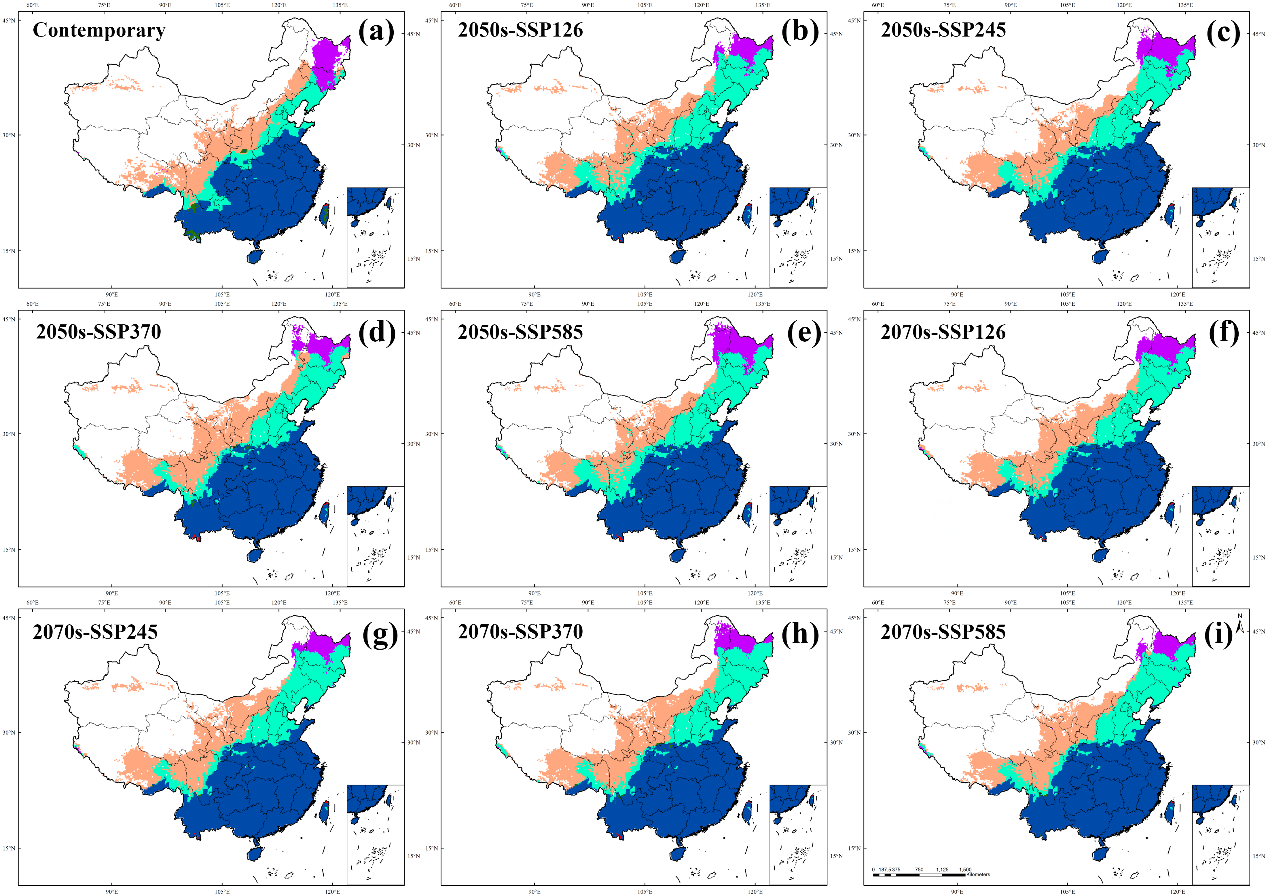


(II) *P. bungeana*

Overlapping range of suitable areas of *P. bungeana* (
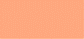
); PWN + *P. bungeana* (
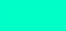
); *M. alternatus* + *P. bungeana* (
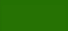
); *M. alternatus* + PWN + *P. bungeana* (
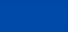
).


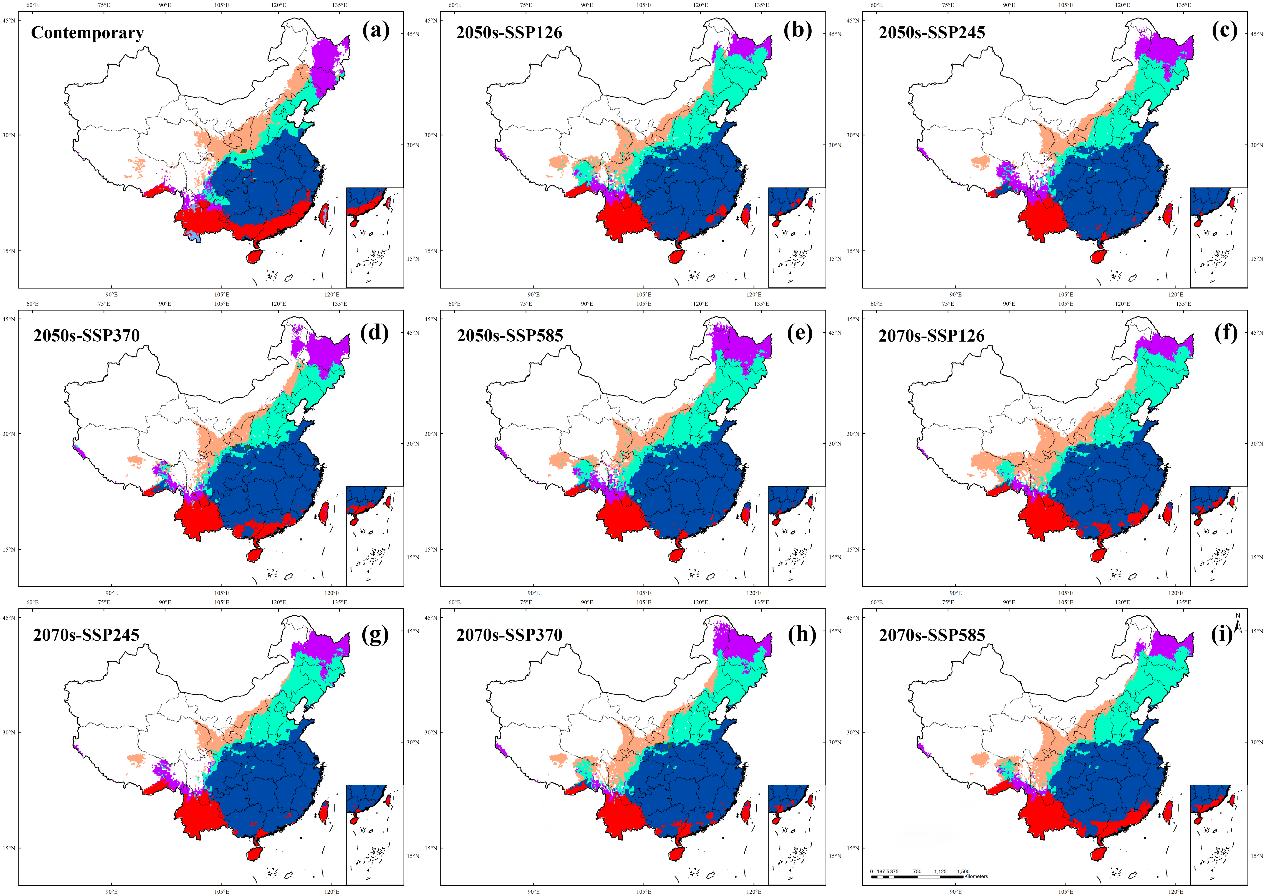


(III) *P. massoniana*

Overlapping range of suitable areas of *P. massoniana* (
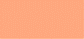
); PWN + *P. massoniana* (
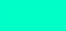
); *M. alternatus* + *P. massoniana* (
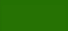
); *M. alternatus* + PWN + *P. massoniana* (
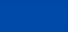
).


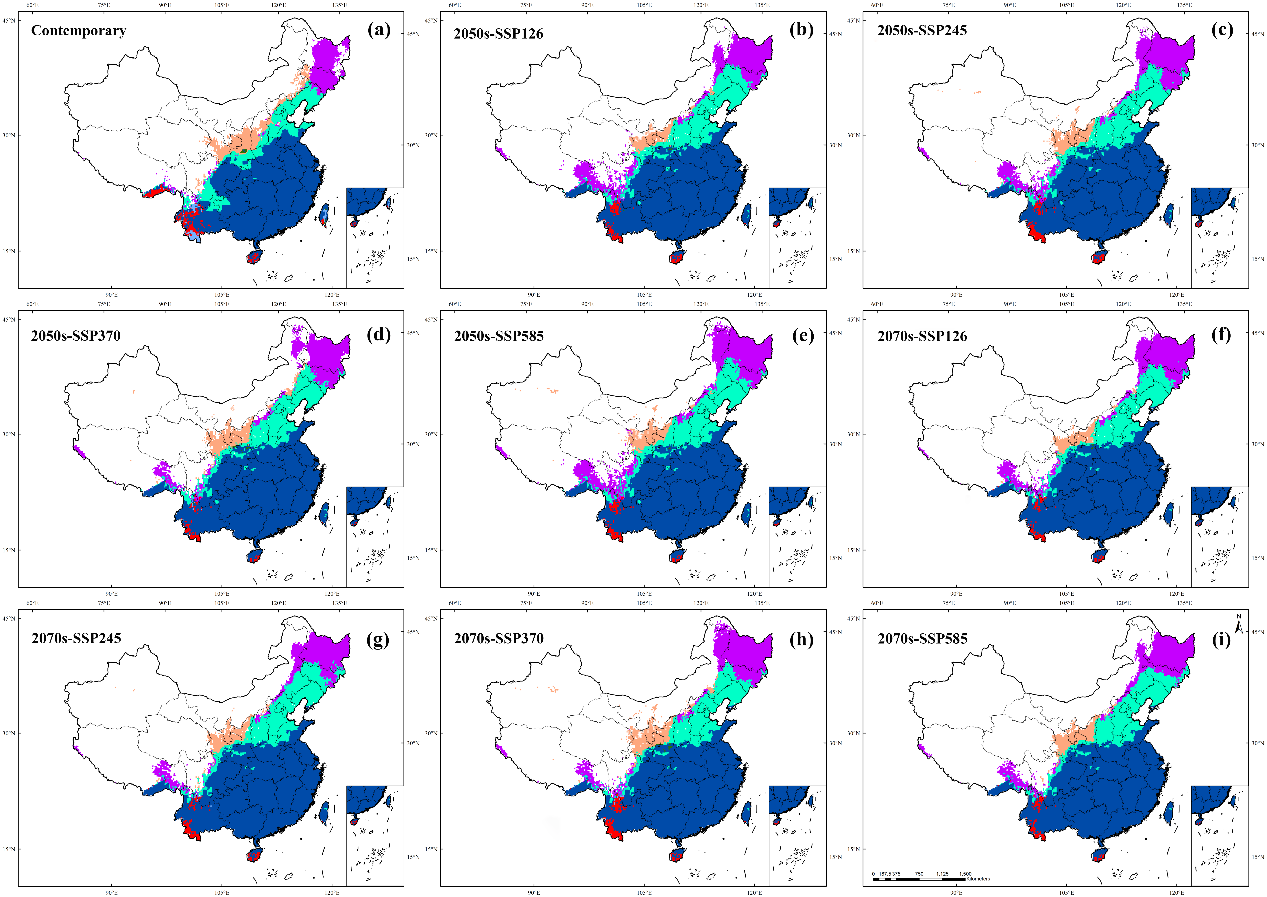


(IV) *P. tabuliformis*

Overlapping range of suitable areas of *P. tabuliformis* (
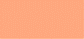
); PWN + *P. tabuliformis* (
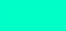
); *M. alternatus* + *P. tabuliformis* (
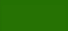
); *M. alternatus* + PWN + *P. tabuliformis* (
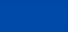
).


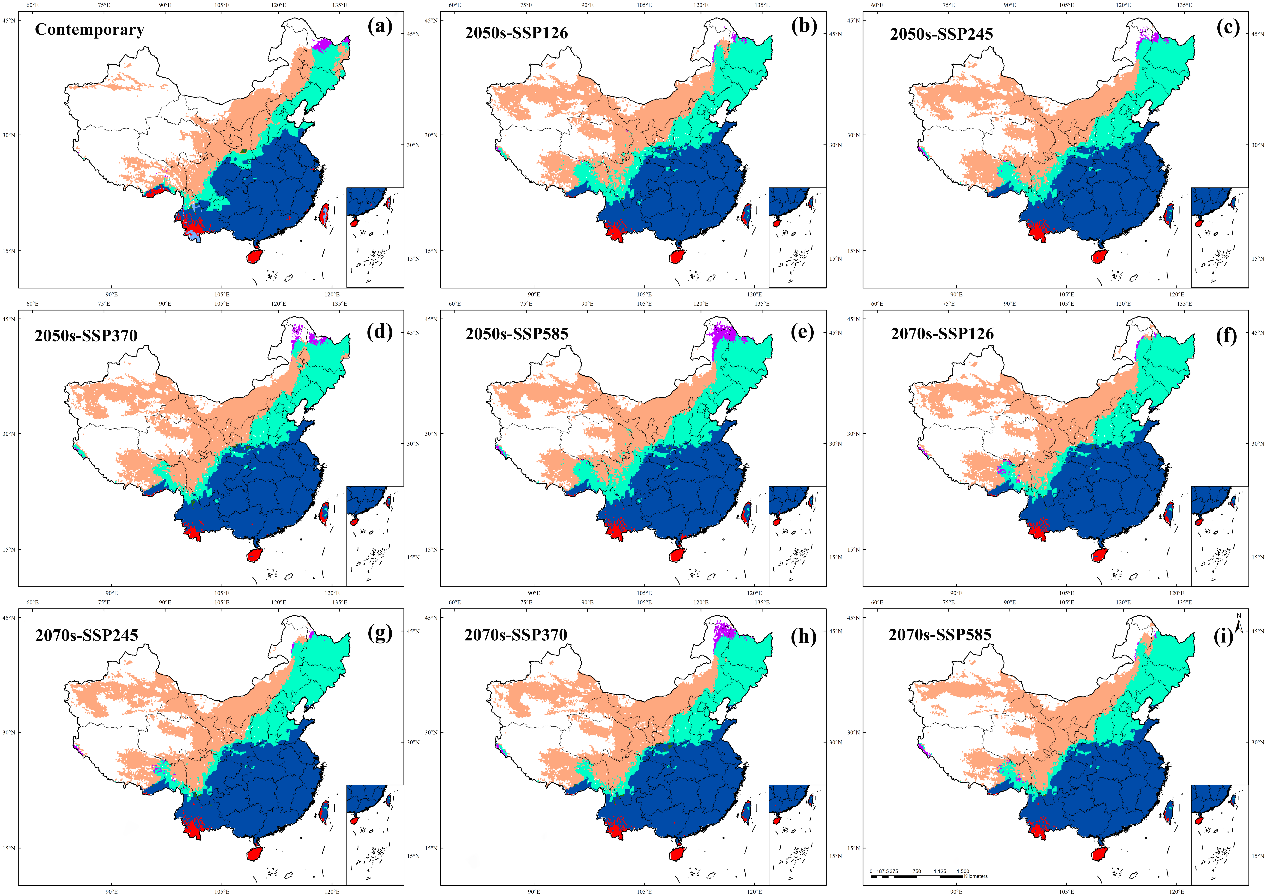


(V) *P. taiwanensis*

Overlapping range of suitable areas of *P. taiwanensis* (
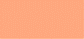
); PWN + *P. taiwanensis* (
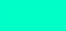
); *M. alternatus* + *P. taiwanensis* (
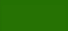
); *M. alternatus* + PWN + *P. taiwanensis* (
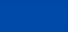
).


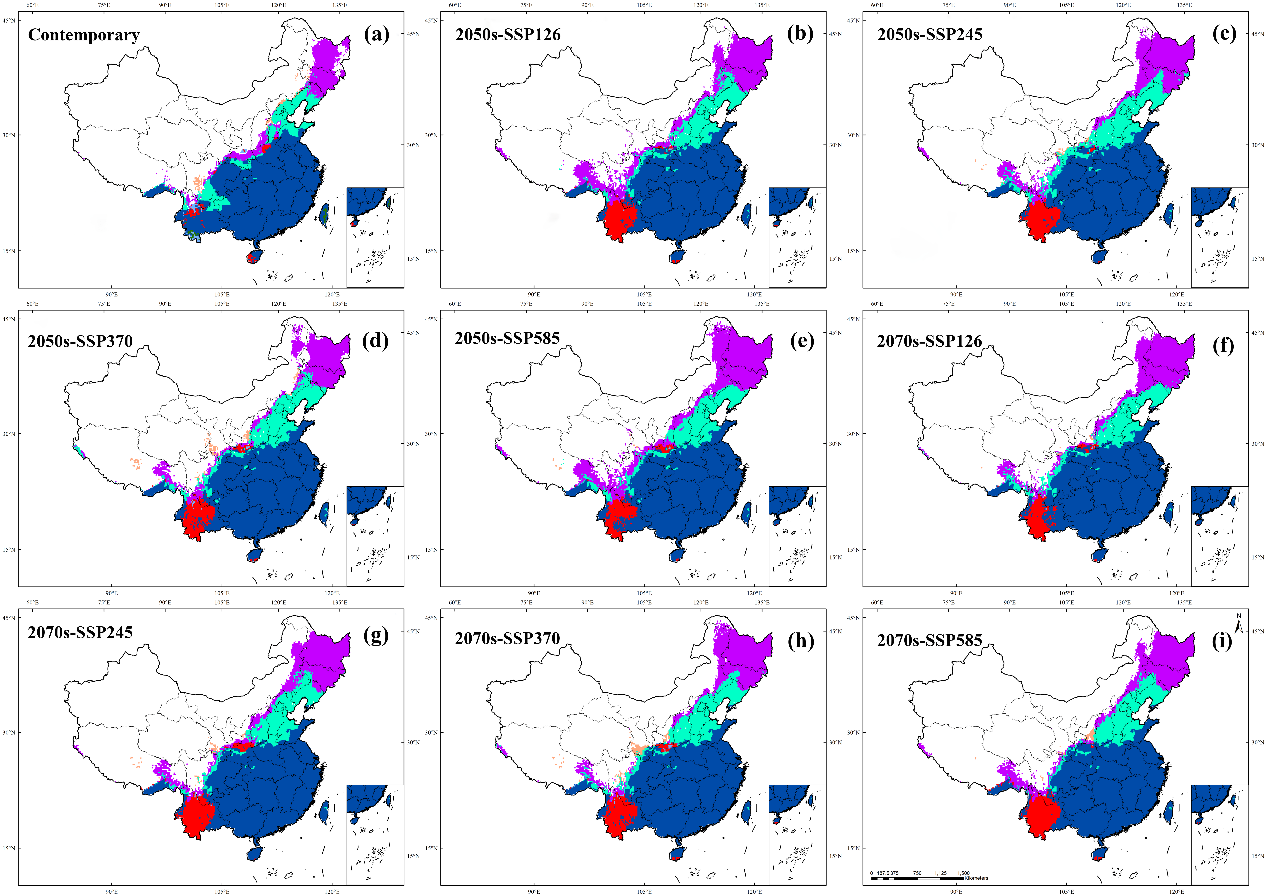


(VI) *P. shurbergia*

Overlapping range of suitable areas of *P. shurbergia* (
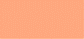
); PWN + *P. shurbergia* (
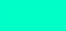
); *M. alternatus* + *P. shurbergia* (
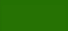
); *M. alternatus* + PWN + *P. shurbergia* (
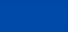
).


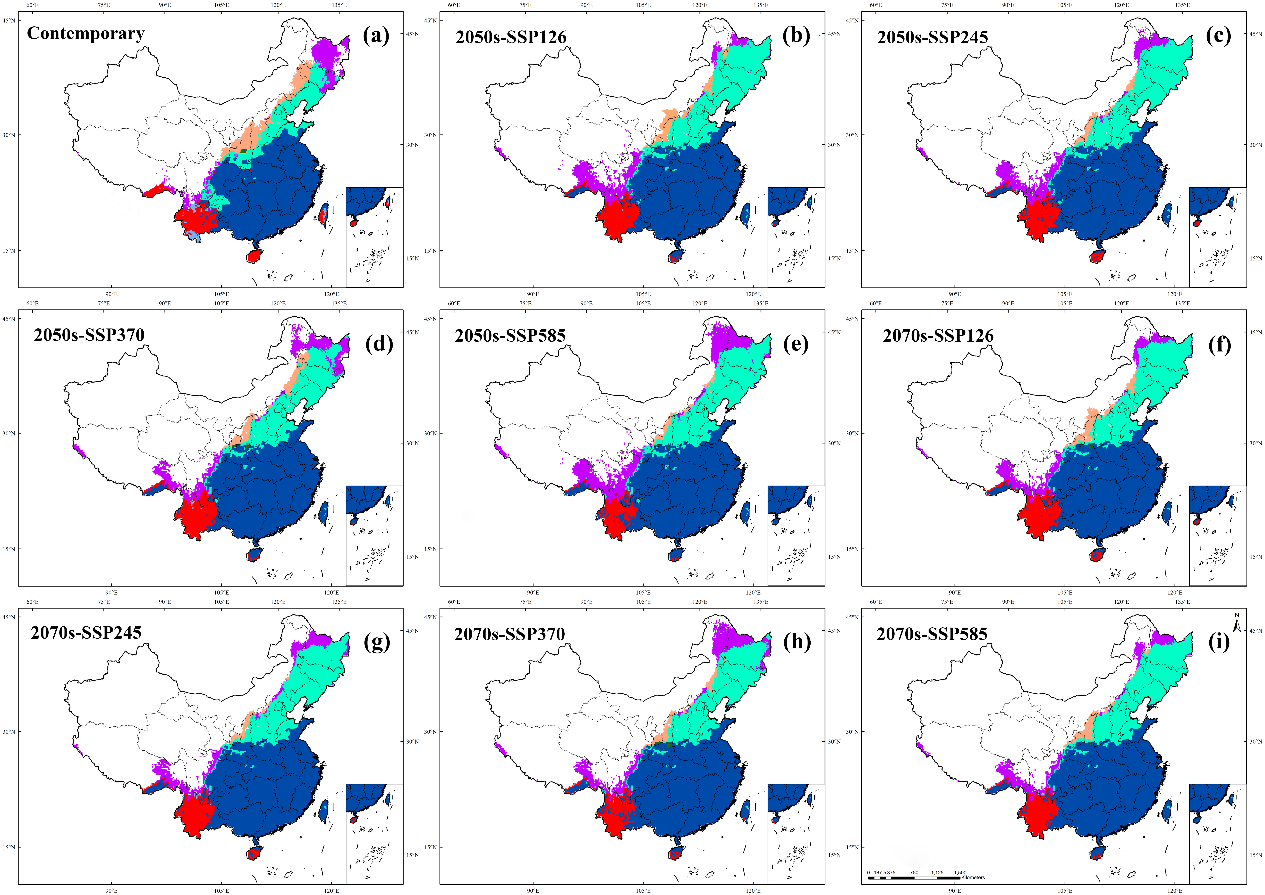


(VII) *P. yunnanensis*

Overlapping range of suitable areas of *P. yunnanensis* (
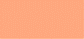
); PWN + *P. yunnanensis* (
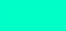
); *M. alternatus* + *P. yunnanensis* (
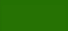
); *M. alternatus* + PWN + *P. yunnanensis* (
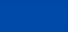
).


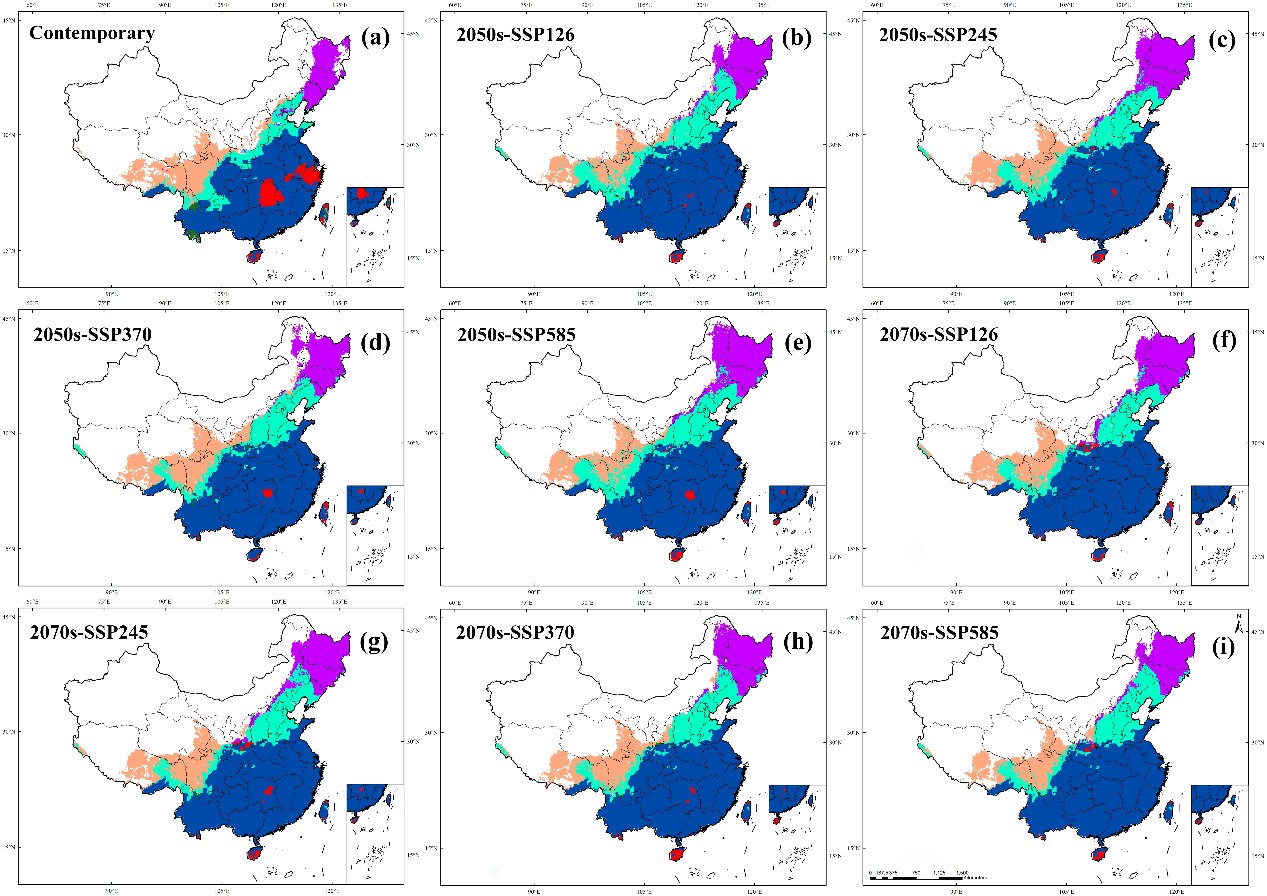


**Table S1.** Bioclimatic variables (https://www.worldclim.org) used in the potential distribution modeling of seven pine species, *Monochamus saltuarius* and pinewood nematode

| **Type** | **Variables** | **Definition** |
| --- | --- | --- |
| 19 Bioclimatic variables | Bio1 | Annual mean temperature (°C) |
|  | Bio2 | Mean diurnal range [Mean of monthly (max temp - min temp)] (°C) |
|  | Bio3 | Isothermality (Bio2/Bio7) (×100) |
|  | Bio4 | Temperature seasonality (standard deviation × 100) (°C) |
|  | Bio5 | maximum temperature in warmest month (°C) |
|  | Bio6 | minimum temperature in coldest month (°C) |
|  | Bio7 | Temperature annual range (Bio5-Bio6) (°C) |
|  | Bio8 | Mean temperature of wettest quarter (°C) |
|  | Bio9 | Mean temperature of driest quarter (°C) |
|  | Bio10 | Mean temperature of warmest quarter (°C) |
|  | Bio11 | Mean temperature of coldest quarter (°C) |
|  | Bio12 | Annual precipitation (mm) |
|  | Bio13 | Precipitation of wettest month (mm) |
|  | Bio14 | Precipitation of driest month (mm) |
|  | Bio15 | Precipitation seasonality (coefficient of variation) |
|  | Bio16 | Precipitation of wettest quarter (mm) |
|  | Bio17 | Precipitation of driest quarter (mm) |
|  | Bio18 | Precipitation of warmest quarter (mm) |
|  | Bio19 | Precipitation of coldest quarter (mm) |

**Table S2.** Key environmental factors used to model potential distribution of the seven pine species, *M. saltuarius* and pinewood nematode

| **Species** | **Bioclimatic variables** |
| --- | --- |
| *P. massoniana* | Bio (2,3,8,10,11,12,15,18,19) |
| *P. taiwanensis* | Bio (1,2,3,6,7,14,15,18) |
| *P. yunnanensis* | Bio (2,3,4,11,15,17,18) |
| *P. armandii* | Bio (2,3,5,7,11,15,18) |
| *P. bungeana* | Bio (2,3,4,8,11,16,19) |
| *P. tabuliformis* | Bio (2,3,4,8,11,15,16) |
| *P. shurbergia*  *M. saltuarius* | Bio (3,4,5,6,8,15,16)  Bio (1,2,3,4,15,18) |
| Pinewood nematode | Bio (2,3,7,8,9,10,13,15,18) |

Refer Table S1 for details of Bioclimatic variables.

**Table S3.** Parameter values for the *Monochmus alternatus* used in the CLIMEX analysis

| Index | Parameter* | Value |
| --- | --- | --- |
| Temperature | DV0 = Limiting low temperature (°C) | 10.8 |
|  | DV1 = Lower optimum temperature (°C) | 15.0 |
|  | DV2 = Upper optimum temperature (°C) | 30.0 |
|  | DV3 = Limiting high temperature (°C) | 33.0 |
|  | PDD = Degree-days (day) | 1690 |
| Moisture | SM0 = Limiting low soil moisture | 0.1 |
|  | SM1 = Lower optimum soil moisture | 0.55 |
|  | SM2 = Upper optimum soil moisture | 1.35 |
|  | SM3 = Limiting high soil moisture | 4.0 |
| Cold stress | TTCS = Temperature threshold (°C) | 8 |
|  | THCS = Stress accumulation rate | -0.00013 |
| Heat stress | TTHS = Temperature threshold (°C) | 33 |
|  | THHS = Stress accumulation rate | 0.0001 |
| Dry stress | SMDS = Threshold soil moisture | 0.25 |
|  | HDS = Stress accumulation rate | -0.001 |
| Wet stress | SMWS = Threshold soil moisture | 4.0 |
|  | HWS = Stress accumulation rate | 0.0001 |

*Parameter values are determined from Song and Xu (2006) and values without units are dimensionless.

**Table S4.** The suitable habitat areas for various species of pine trees and PWN in China under various scenarios of climate change

| The suitable habitat areas for various species of pine trees and PWN in China under various climatic scenarios （×10^4^ km^2^） | | | | | | | | | | |
| --- | --- | --- | --- | --- | --- | --- | --- | --- | --- | --- |
| Species | suitability | Current | 2050s | | | | 2070s | | | |
|  |  |  | SSP126 | SSP245 | SSP370 | SSP585 | SSP126 | SSP245 | SSP370 | SSP585 |
| *P. massoniana* | Low | 73.9 | 85.6 | 80.4 | 83.8 | 89.0 | 83.3 | 87.2 | 82.3 | 86.3 |
|  | moderate | 65.5 | 53.3 | 70.7 | 69.7 | 67.1 | 50.1 | 53.9 | 68.1 | 50.2 |
|  | high | 180 | 222 | 213 | 208 | 214 | 225 | 231 | 216 | 229 |
|  | total | 319 | 361 | 364 | 361 | 370 | 358 | 372 | 366 | 365 |
| *P. taiwanensis* | Low | 71.1 | 56.2 | 60.6 | 57.0 | 53.4 | 54.6 | 53.9 | 59.3 | 52.0 |
|  | moderate | 91.9 | 59.0 | 59.0 | 67.3 | 51.7 | 43.0 | 64.6 | 66.4 | 45.6 |
|  | high | 115 | 186 | 196 | 185 | 197 | 208 | 182 | 188 | 202 |
|  | total | 278 | 301 | 315 | 309 | 302 | 306 | 301 | 314 | 300 |
| *P. armandii* | Low | 86.0 | 133 | 122 | 126 | 123 | 124 | 140 | 136 | 125 |
|  | moderate | 134 | 130 | 131 | 128 | 138 | 132 | 130 | 134 | 129 |
|  | high | 216 | 278 | 276 | 293 | 270 | 277 | 279 | 292 | 281 |
|  | total | 436 | 541 | 529 | 548 | 530 | 533 | 548 | 562 | 535 |
| *P.bungeana* | Low | 86.2 | 145 | 114 | 114 | 139 | 162 | 107 | 152 | 138 |
|  | moderate | 84.2 | 95.4 | 91.5 | 72.3 | 83.1 | 99.1 | 87.7 | 78.7 | 89.1 |
|  | high | 127 | 181 | 177 | 173 | 183 | 167 | 179 | 170 | 167 |
|  | total | 297 | 421 | 382 | 359 | 405 | 429 | 373 | 401 | 394 |
| *P. tabuliformis* | Low | 117 | 180 | 199 | 202 | 198 | 192 | 199 | 206 | 179 |
|  | moderate | 171 | 237 | 193 | 197 | 195 | 180 | 185 | 206 | 182 |
|  | high | 237 | 282 | 302 | 290 | 294 | 310 | 298 | 281 | 301 |
|  | total | 526 | 699 | 694 | 689 | 687 | 683 | 683 | 693 | 663 |
| *P. shurbergia* | Low | 63.3 | 82.1 | 81.9 | 74.3 | 87.4 | 83.3 | 82.3 | 89.3 | 77.2 |
|  | moderate | 66.4 | 76.7 | 74.3 | 53.1 | 54.7 | 74.2 | 64.9 | 54.1 | 65.6 |
|  | high | 182 | 225 | 221 | 224 | 231 | 224 | 224 | 225 | 233 |
|  | total | 311 | 384 | 377 | 351 | 373 | 382 | 371 | 369 | 376 |
| *P. yunnanensis* | Low | 122 | 114 | 105 | 106 | 108 | 95.3 | 110 | 98.3 | 97.8 |
|  | moderate | 102 | 176 | 194 | 188 | 171 | 179 | 170 | 185 | 172 |
|  | high | 116 | 129 | 115 | 127 | 137 | 129 | 129 | 136 | 142 |
|  | total | 340 | 419 | 414 | 420 | 416 | 404 | 409 | 420 | 411 |
| PWN | Low | 87.6 | 127 | 116 | 120 | 120 | 138 | 131 | 119 | 121 |
|  | moderate | 55.7 | 84.8 | 97.8 | 61.5 | 109.8 | 67.9 | 73.9 | 83.7 | 80.0 |
|  | high | 197 | 228 | 231 | 231 | 231 | 227 | 227 | 233 | 226 |
|  | total | 340 | 440 | 444 | 413 | 461 | 432 | 433 | 436 | 426 |
